# Metagenomic analysis revealed the potential role of gut microbiome in gout

**DOI:** 10.1038/s41522-021-00235-2

**Published:** 2021-08-09

**Authors:** Yongliang Chu, Silong Sun, Yufen Huang, Qiang Gao, Xuefeng Xie, Peng Wang, Junxia Li, Lifeng Liang, Xiaohong He, Yiqi Jiang, Maojie Wang, Jianhua Yang, Xiumin Chen, Chu Zhou, Yue Zhao, Fen Ding, Yi Zhang, Xiaodong Wu, Xueyuan Bai, Jiaqi Wu, Xia Wei, Xianghong Chen, Zhen Yue, Xiaodong Fang, Qingchun Huang, Zhang Wang, Runyue Huang

**Affiliations:** 1 State Key Laboratory of Dampness Syndrome of Chinese Medicine, The Second Affiliated Hospital of Guangzhou University of Chinese Medicine (Guangdong Provincial Hospital of Chinese Medicine), Guangzhou, China; 2 Zhuhai Branch, Guangdong Provincial Hospital of Chinese Medicine, Zhuhai, China; 3 BGI-Shenzhen, Shenzhen, China; 4 BGI Genomics, BGI-Shenzhen, Shenzhen, China; 5 BGI Institute of Applied Agriculture, BGI-Shenzhen, Shenzhen, China; 6 Department of Computer Science, City University of Hong Kong, Hong Kong, China; 7 Guangdong Provincial Key Laboratory of Clinical Research on Traditional Chinese Medicine Syndrome, Guangzhou, China; 8 Center for Molecular Medicine, University Medical Center Utrecht, Utrecht, Netherlands; 9 Guangdong-Hong Kong-Macau Joint lab on Chinese Medicine and Immune Disease Research, Guangzhou University of Chinese Medicine, Guangzhou, China; 10 School of Environmental Science and Engineering, Sun Yat-Sen University, Guangzhou, China; 11 Institute of Ecological Sciences, School of Life Sciences, South China Normal University, Guangzhou, China

**Keywords:** Microbiota, Metagenomics

## Abstract

Emerging evidence indicates an association between gut microbiome and arthritis diseases including gout. However, how and which gut bacteria affect host urate degradation and inflammation in gout remains unclear. Here we performed a metagenome analysis on 307 fecal samples from 102 gout patients and 86 healthy controls. Gout metagenomes significantly differed from those of healthy controls. The relative abundances of *Prevotella*, *Fusobacterium*, and *Bacteroides* were increased in gout, whereas those of *Enterobacteriaceae* and butyrate-producing species were decreased. Functionally, gout patients had greater abundances for genes in fructose, mannose metabolism and lipid A biosynthesis, and lower for genes in urate degradation and short chain fatty acid production. A three-pronged association between metagenomic species, functions and clinical parameters revealed that decreased abundances of species in *Enterobacteriaceae* were associated with reduced amino acid metabolism and environmental sensing, which together contribute to increased serum uric acid and C-reactive protein levels in gout. A random forest classifier based on three gut microbial genes showed high predictivity for gout in both discovery and validation cohorts (0.91 and 0.80 accuracy), with high specificity in the context of other chronic disorders. Longitudinal analysis showed that uric-acid-lowering and anti-inflammatory drugs partially restored gut microbiota after 24-week treatment. Comparative analysis with obesity, type 2 diabetes, ankylosing spondylitis and rheumatoid arthritis indicated that gout metagenomes were more similar to those of autoimmune than metabolic diseases. Our results suggest that gut dysbiosis was associated with dysregulated host urate degradation and systemic inflammation and may be used as non-invasive diagnostic markers for gout.

## Introduction

Gout is an inflammatory arthritis disease that primarily involves the joints and is considered a risk factor for hypertension and cardiovascular disease^1^. Gout is more common in males than females^2^, with a rise in prevalence due to changes in diet and lifestyle^3^. The reported prevalence of gout in the US was 3.9% in 2015–2016^4^, while in European countries it was ranged between 1 and 4% from 2003 to 2014^5^. A meta-analysis of 30 studies revealed the prevalence of gout in mainland China was ~1.1% from 2000 to 2016. Despite a relatively lower prevalence of gout in China, it shows an ascending trend by year^6^.

Gout is known to be primarily caused by an abnormal increase in uric acid and the crystallization of monosodium urate (MSU) crystals. In healthy people, there are two routes to excrete uric acid from the body: the kidney, which is responsible for discharging 70% of uric acid, and the intestine, which excretes the remaining 30%^7^. Recent studies have shown that the gut microbiota dysbiosis is related to arthritis diseases, including ankylosing spondylitis (AS)^8,9^, rheumatoid arthritis (RA)^10,11^ and psoriasis^12,13^, yet the association between gut microbiota and gout remains poorly characterized. A previous study showed the abundance of *Bacteroides caccae* and *B. xylanisolvens* was significantly enriched in gout patients, whereas the abundance of *Faecalibacterium prausnitzii* and *Bifidobacterium pseudocatenulatum* was decreased^14^. Gut microbial functions including purine metabolism were also altered in gout patients^14^. Another study combining microbiome and metabolome showed opportunistic pathogens in *Bacteroides*, *Porphyromonadaceae*, *Rhodococcus*, *Erysipelatoclostridium*, and *Anaerolineaceae* were increased, along with altered metabolites in uric acid excretion and purine metabolism in gout patients^15^. Although insightful, most of these studies had small sample size without or with only a few samples for independent validation so their generalizability was limited. Additionally, the interactions between gut microbiota and gout-associated clinical parameters, and the response of gut microbiota to therapeutic interventions in gout remained unexplored. Additionally, the relationship between gut microbiota and key gout-associated clinical parameters, and the response of gout microbiota to therapeutic interventions remained unexplored. As such, a comprehensive view of how and which gut microbes may impact the key pathophysiological processes such as host urate degradation and systemic inflammation in gout is lacking, and warrants investigation in further longitudinal studies with detailed characterization of patient clinical parameters.

Here we conducted a large-scale metagenomic analysis on the gut microbiome of 307 samples collected longitudinally from gout patients and healthy controls. Utilizing both discovery and validation cohorts, we sought to investigate the taxonomic and functional signatures of gut microbiome in gout, its association with gout-related clinical parameters such as serum uric acid (SUA) and inflammation, its response to therapeutic intervention, and the gout-related microbiome signatures in relation to the broader autoimmune and metabolic disorders.

## Results and discussion

### Overview of gut metagenome in gout patients and controls

We performed metagenomic shotgun sequencing for 307 fecal samples collected from 188 individuals. At baseline, there were 140 samples in discovery cohort collected during 2016–2017, including 77 gout patients and 63 healthy controls. There were 48 samples in validation cohort collected in the year of 2018, consisting of 25 gout patients and 23 healthy controls. In addition, 70, 40, and 9 fecal samples were collected from gout patients in discovery cohort longitudinally at three follow-up time points (week 2, 4, and 24 post-baseline), to assess the effects of therapeutic intervention on the gut microbiome in gout patients. All patients’ information is summarized in Supplementary Data 1. Compared with controls, gout patients had elevated levels of inflammation and renal indices, including C-reactive protein (CRP), erythrocyte sedimentation rate (ESR), SUA, and serum creatinine (SCr) (Table 1). There was a higher body mass index (BMI) in gout patients, in accordance with the view that gout was associated with obesity^16^. In addition, CRP, ESR, SUA, and other gout-associated indices including visual analog scale (VAS), arthralgia, joint swelling scores and joint tenderness scores declined in gout patients following treatment (Supplementary Data 1).

**Table 1:** The baseline characteristics of subjects in discovery and validation cohorts.

Characteristics^a^	Discovery control (*n* = 63)	Discovery gout (*n* = 77)	Discovery *P* value^b^	Validation control (*n* = 25)	Validation gout (*n* = 23)	Validation *P* value^b^	Total (*n* = 188)
Age, years	40.0 ± 12.1	39.9 ± 12.9	0.965	38.3 ± 13.6	41.9 ± 14.4	0.227	40.0 ± 12.9
BMI, kg/m^2^	23.5 ± 3.0	25.0 ± 2.5	<0.001	22.4 ± 2.3	25.8 ± 2.9	<0.001	24.3 ± 2.9
CRP, mmol/L	2.0 ± 3.2	24.5 ± 29.9	<0.001	1.5 ± 2.1	21.8 ± 18.5	<0.001	14.5 ± 23.6
eGFR, ml/min	99.7 ± 15.4	90.6 ± 22.8	0.01	104.7 ± 20.6	96.5 ± 20.8	0.286	95.9 ± 20.8
ESR, mm/h	8.8 ± 7.5	30.4 ± 24.6	<0.001	12.7 ± 11.7	32.9 ± 15.4	<0.001	21.9 ± 20.9
SCr, μmol/L	82.3 ± 12.2	95.1 ± 21.2	<0.001	83.7 ± 16.0	88.5 ± 22.3	0.502	88.7 ± 19.0
SUA, μmol/L	361.4 ± 50.4	539.2 ± 118.7	<0.001	350.8 ± 57.8	555.2 ± 156.8	<0.001	458.7 ± 136.6
Urea, mmol/L	5.0 ± 1.1	4.8 ± 1.5	0.129	5.0 ± 1.3	4.6 ± 1.7	0.149	4.9 ± 1.4

^a^data are mean ± s.d; *BMI* body mass index, *CRP* C-reactive protein, *eGFR* estimated glomerular filtration rate, *ESR* erythrocyte sedimentation rate, *SCr* serum creatinine, *SUA* serum uric acid.

^b^*P* values were obtained by Wilcoxon rank-sum test.

**Fig. 1:**
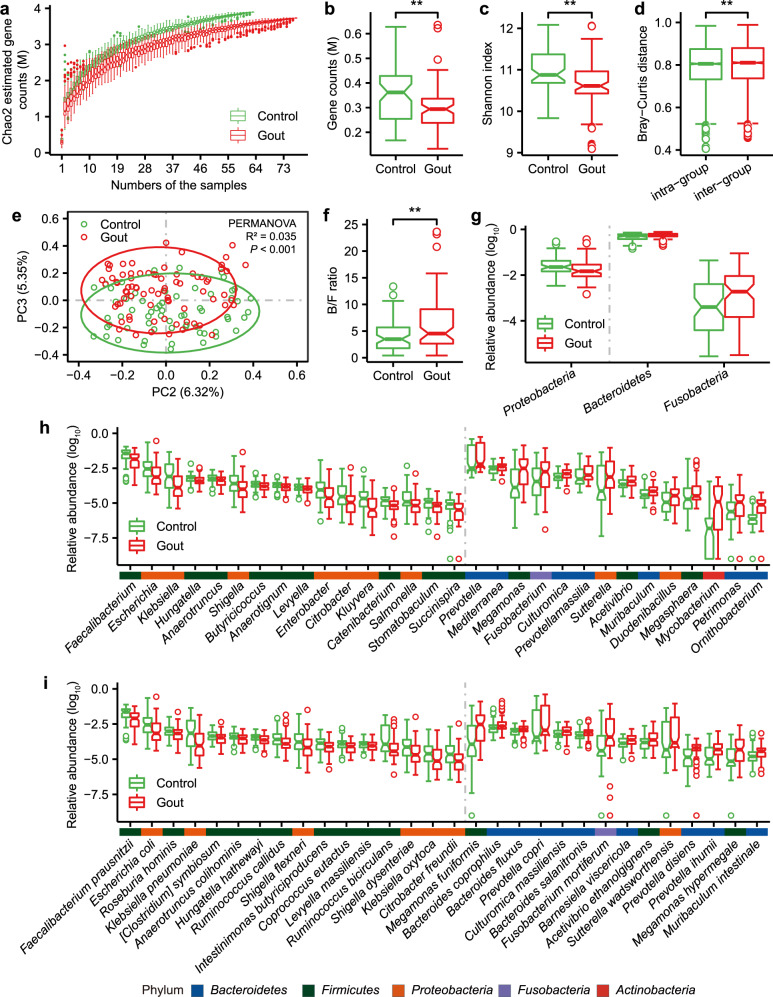
Gut microbial alterations in gout patients. **a** The gene rarefaction curves based on the Chao2 estimated gene counts in healthy controls (*n* = 63) and gout patients (*n* = 77) (paired Wilcoxon rank-sum test for median gene counts *P* = 5.6e–12). **b** Box and whisker plot of gene count in the healthy controls and gout patients. Wilcoxon rank-sum test was used to determine significance. **P < 0.01. **c**, **d** Box and whisker plots of alpha diversity (Shannon index) and beta diversity (Bray–Curtis distance) at the gene level. Wilcoxon rank-sum test was used to determine significance. **P < 0.01. To exclude the influence of the various data sizes among the samples, panels **a**, **b**, **d** and **d** were based on 11 M matched reads per individual. **e** Principal component analysis (PCA) based on the gene relative abundance profile. The 95% confidence ellipses were shown for gout and control samples. **f** The Bacteroidetes/Firmicutes ratio (Wilcoxon rank-sum test; **P < 0.01). The relative abundance of differential phyla (**g** top 3), genera (**h** top 30) and species (**i** top 30) between gout patient and healthy control groups (FDR *P* < 0.05, Wilcoxon rank-sum test). The color bar above genera or species names were colored according to the phylum. For all box and whisker plots, the center line represents median. The bounds of box represent the first and third quartiles. The upper whisker extends from the hinge to the largest value no further than 1.5 * interquartile range (IQR) from the hinge. The lower whisker extends from the hinge to the smallest value at most 1.5 * IQR of the hinge. The notch represents a confidence interval around the median as the median ± 1.58*IQR/sqrt(*n*).

Consistent with previous findings^15^, gout patients had lower microbial gene richness and diversity compared to healthy controls both in discovery (as shown in rarefaction analysis [paired Wilcoxon rank-sum test for median gene counts: *P* = 5.6e–12, Fig. 1a], the actual number of genes observed: [gene number, Wilcoxon rank-sum test, *P* = 0.0024, Fig. 1b], and alpha diversity: [Shannon index, Wilcoxon rank sum-test, *P* = 0.0016, Fig. 1c]) and validation (gene number, Wilcoxon rank-sum test, *P* = 0.016; Shannon index, Wilcoxon rank-sum test, *P* = 0.091; Supplementary Fig. 1a, b) cohorts. Beta-diversity analysis showed a small, significant increase in dissimilarity in microbial community between samples in gout and control groups than those within each group (discovery cohort: *P* = 0.0069, mean: 0.796 vs 0.803, median: 0.805 versus 0.810, Fig. 1d; validation cohort: *P* = 1.618e–11, mean: 0.541 vs 0.565, median: 0.536 versus 0.560, Supplementary Fig. 1c). Considering that enterotype was reported to be disease-related^17,18^, we examined enterotype of all samples in discovery cohort (Supplementary Fig. 2a). Enterotype was a main factor explaining the variability of the gut microbial composition of samples (PERMANOVA *P* < 1e–04, *R*^2^ = 0.046) and the first principal coordinate (PC) was significantly associated with enterotype (Wilcoxon rank-sum test, *P* = 2.16e–20). However, no significant difference in enterotype distribution was observed between gout patients and controls (*P* = 0.8541, Fisher’s exact test; Supplementary Fig. 2b). When samples were ordinated by the second and third PCs, there was a significant association between gut microbial composition and gout both in discovery (PERMANOVA, *P* < 0.001, *R*^2^ = 0.035; Fig. 1e) and validation (PERMANOVA, *P* < 0.001, *R*^2^ = 0.063; Supplementary Fig. 1d) cohorts. And the third PC was significantly associated with gout in discovery cohort (Wilcoxon rank-sum test, *P* = 2.01e–07). These results suggested that when enterotype was accounted for, disease status became one major factor associated with gut microbiota. Among all demographic factors (age, BMI, alcohol drinking, probiotics use, vegetarian diet) that may influence gut microbiome, only BMI was significantly different between gout patients and healthy controls so it was included as a confounder in statistical analyses. The overall gut microbiome was not significantly associated with BMI using PERMANOVA (*P* = 0.2006, *R*^2^ = 0.008). MaAsLin analysis using all demographic factors indicated that *Geodermatophilus* was associated with BMI, *Paraburkholderia* and *Histophilus* were associated with alcohol drinking, and *Varibaculum*, *Dakarella*, and *Suterrella* were associated with probiotics (FDR *P* < 0.25). Among them, only *Sutterella* was differentially abundant between gout patients and controls (FDR *P* < 0.05), suggesting its association with gout may be attributed to probiotic use.

### Taxonomic alternation of gut metagenome in gout

Next, we identified gut microbial taxa associated with gout. In discovery cohort (*n* = 140), a total of 18 phyla, 415 genera and 861 species were identified (Supplementary Fig. 3a, b and Supplementary Data 1). At the phylum level, *Bacteroidetes* (FDR *P* = 0.022) and *Fusobacteria* (FDR *P* = 0.0183) were increased in gout patients, whereas *Proteobacteria* (FDR *P* = 0.0183) was decreased (Fig. 1g). The ratio of *Bacteroidetes* to *Firmicutes* was higher in gout patients (*P* = 0.0098; Fig. 1f). 110 genera and 223 species were significantly different in abundance between gout patients and controls (FDR *P* < 0.05; Fig. 1h, i and Supplementary Data 1). All differential species in *Bacteroides* (3 species), *Prevotella* (13 species), and *Fusobacterium* (4 species) were enriched in gout patients. *Bacteroides* was reported to be enriched in the gut microbiota of gout patients^14^. *P. copri* (FDR *P* = 0.0169) was increased in RA and was pro-inflammatory in a mouse model of colitis^19^. *F. nucleatum* (FDR *P* = 0.0035) was also pro-inflammatory and conducive to the development of colorectal cancer^20^. In comparison, several butyrate-producing species, such as *Roseburia* spp. (3 species), *Coprococcus* spp. (3 species), *Eubacterium* spp. (3 species), *F. prausnitzii* and butyrate-producing bacterium SS3/4 were enriched in healthy controls; many of these species were reported with potential anti-inflammatory effects^21,22^. In addition, members of *Enterobacteriaceae* reported to degrade uric acid^23^, such as *Escherichia* spp. (2 species), *Klebsiella* spp. (7 species), *Enterobacter* spp. (9 species) and *Citrobacter* spp. (7 species), were enriched in healthy controls (FDR *P* < 0.05), possibly helping reduce uric acid accumulation in healthy individuals. 128 out of 223 significantly different species were validated in validation cohort (Supplementary Fig. 1e). As a complement to the gene-based taxonomic profiling, we performed reads-based taxonomic profiling using MetaPhlAn3^24^, which identified 12 phyla, 194 genera, and 485 species. Of them, 8 phyla, 29 genera and 43 species were associated with gout (FDR *P* < 0.25, Supplementary Fig. 4, Supplementary Data 1), including key taxa such as *Faecalibacterium*, *Enterobacter*, *Klebsiella*, *Prevotella*, *Fusobacterium* and *Escherichia* as described above.

### Functional alternation of gut metagenome in gout

Principal component analysis (PCA) revealed significantly altered gut microbial functional profiles in gout patients compared to controls (PERMANOVA, *P* < 0.001, *R*^2^ = 0.036; PC1, *P* = 0.0008; PC2, *P* = 0.0043; Supplementary Fig. 5a, b). In total, 2666 out of 7289 KEGG Orthologs (KOs) were differentially abundant between gout patients and controls (2260 and 406 KOs enriched in controls and gout patients, respectively; FDR *P* < 0.05; Supplementary Data 1). This corresponded to 40 KEGG pathways and 129 KEGG modules that showed significant difference according to the reporter score (|reporter score| > 1.65) in discovery cohort (Supplementary Fig. 5c, d and Supplementary Data 1) and 9 of these pathways were significant in validation cohort (Supplementary Fig. 1f). Overall, gout patients had higher abundance of carbohydrate metabolism (4 pathways), energy metabolism (3 pathways), metabolism of terpenoids and polyketides (3 pathways) and biosynthesis of other secondary metabolites (3 pathways), whereas healthy controls were enriched in pathways in cell motility (2 pathways) and xenobiotics biodegradation and metabolism (2 pathways).

**Fig. 2:**
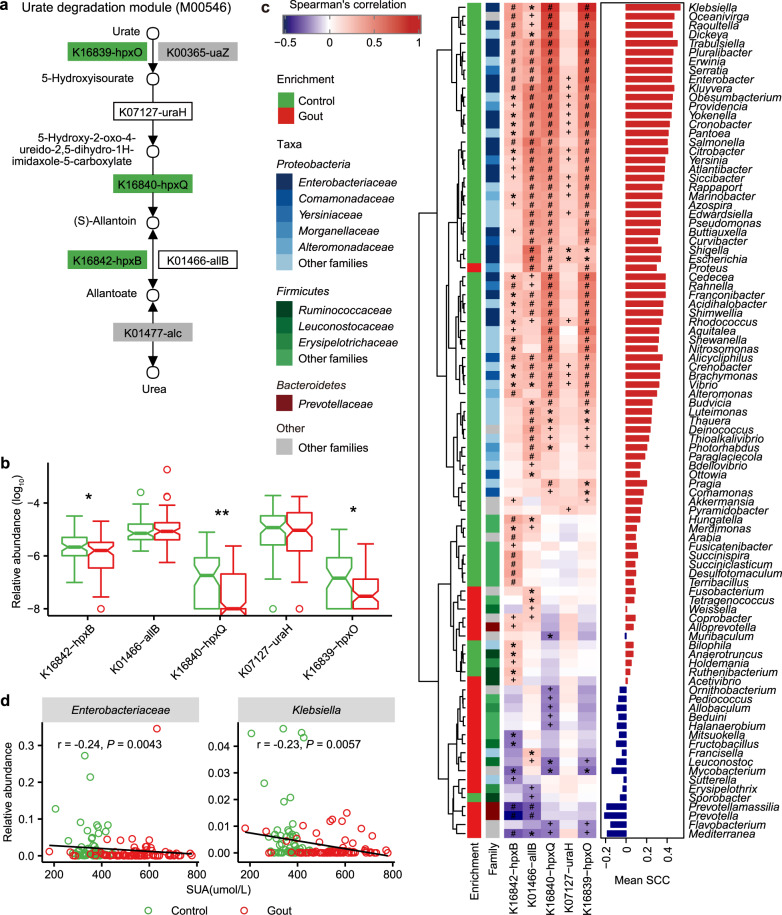
Gout-associated microbial gene functions related to urate degradation. **a** KEGG module for urate degradation. **b** Relative abundance of KOs involved in urate degradation. Significantly enriched KOs were identified by Wilcoxon rank-sum test, and the boxes or KO names were colored according to the direction of enrichment. Green, enriched in healthy controls (FDR *P* < 0.05). Boxes with no color or KO names with black, no difference; boxes with gray, not detected in samples. **c** Correlations between gout-associated genera and urate degradation-associated KOs (red and purple for positive and negative correlation, respectively). Spearman correlation test: ‘plus’ denotes FDR *P* < 0.05; ‘asterisk’ denotes FDR *P* < 0.01; ‘hash’ denotes FDR *P* < 0.001. The enrichment direction and family classification of genera were shown in left panel and the mean Spearman’s correlation coefficient of each genus with urate degradation-associated KOs was shown in the right panel. **d** The associations between SUA and *Enterobacteriaceae* or *Klebsiella*. Spearman’s rank correlation was calculated by taking the species relative abundance and SUA content. An inverse correlation was observed between SUA and *Enterobacteriaceae* and *Klebsiella*. For all box and whisker plots, the center line represents median. The bounds of box represent the first and third quartiles. The upper whisker extends from the hinge to the largest value no further than 1.5 * interquartile range (IQR) from the hinge. The lower whisker extends from the hinge to the smallest value at most 1.5 * IQR of the hinge. The notch represents a confidence interval around the median as the median ± 1.58*IQR/sqrt(*n*). hpxO FAD-dependent urate hydroxylase, uraH 5-hydroxyisourate hydrolase, hpxQ 2-oxo-4-hydroxy-4-carboxy-5-ureidoimidazoline decarboxylase, allB and hpxB allantoinase.

Previous studies suggested that gut microbiota was involved in dysregulated urate degradation in gout patients^14,15^. We observed that healthy controls had a higher abundance for genes in urate degradation (FDR *P* < 0.05; Fig. 2a, b) and the major microbial contributor was *Enterobacteriaceae* spp. (FDR *P* < 0.05, Spearman’s rank correlation; Fig. 2c and Supplementary Data 1) in which *Klebsiella* showed the highest correlations with the urate degradation KOs (mean Spearman’s rank correlation = 0.53). In addition, SUA was negatively correlated with *Enterobacteriaceae* (*r* = −0.24, *P* = 0.0043, Spearman’ rank correlation) and *Klebsiella* (*r* = −0.23, *P* = 0.0057, Spearman’ rank correlation; Fig. 2d), supporting that members of *Enterobacteriaceae* may contribute to uric acid degradation. Despite with the same trends, these correlations were not statistically significant within gout or healthy individuals (Supplementary Fig. 6). Previous study showed that the ability to degrade uric acid as a source of nitrogen and carbon was widely distributed among the *Enterobacteriaceae* species^23,25^. Correspondingly, we profiled the distribution of KOs in urate degradation pathways among 1135 complete bacterial genomes in KEGG (Supplementary Fig. 7 and Supplementary Data 1). *Proteobacteria* (706 species) was the predominant contributor, within which the largest family with urate-degrading KOs was *Enterobacteriaceae* (119 species). These results suggested that *Enterobacteriaceae* spp. may be involved in uric acid degradation in the gut and maintain the stability of uric acid in human body.

Dietary fiber can be fermented by the gut microbiota to generate SCFAs. Other than the chronic inflammatory diseases such as inflammatory bowel disease^26^, asthma^27^, and allergies^28^ in which SCFAs were extensively studied, SCFAs may also exert influence on the inflammation in gout. For example, butyrate was reported to be able to suppress acute gout arthritis by inhibiting histone deacetylases and decrease MSU-induced production of IL-1β, IL-6, and IL-8^29^. Acetate was also shown to promote resolution of the inflammatory response induced by MSU in a mouse model of gout^30^. As SCFA-producing species was enriched in healthy controls in our cohorts, we analyzed the abundance of genes encoding key enzymes in SCFA production and found that the relative abundance of genes responsible for propionate and butyrate biosynthesis were higher in healthy controls, whereas genes for acetate biosynthesis did not show significant differences (Supplementary Fig. 8).

LPS is reported to play a crucial role in immune homeostasis^31^ and may be involved in gout-associated inflammation^32^. We observed enrichment of genes in LPS biosynthesis and lipid A biosynthesis in gout patients (Supplementary Fig. 9a, b). The major contributors to lipid A biosynthesis were *Bacteroides* and *Prevotella*, both enriched in gout patients. Conversely, the lipid A biosynthesis genes from *Proteobacteria* (FDR *P* = 0.017) species, including *E. coli* (FDR *P* = 0.005) and *K. pneumoniae* (FDR *P* = 0.014), were elevated in healthy controls (Supplementary Fig. 9c). It has been recently suggested that lipid A produced from *Enterobacteriaceae* have six acyl chains and may be a beneficial innate immune activator, whereas lipid A produced by *Bacteroides* and *Prevotella* are structurally distinct (i.e. with four or five acyl chain) and may inhibit innate immune activation and endotoxin tolerance^33,34^. Therefore, it is tempting to speculate that there was a turnover of lipid A biosynthesis between these two different isoforms that contributed to the inflammation in gout. In support, *E. coli* was reported to suppress arthritis through LPS^35,36^. *Klebsiella* spp. were also shown to be depleted in autoimmune diseases, including RA^10^ and AS^8,9^.

### Gut metagenome associated with gout clinical parameters

Next we sought to understand which gut microbial taxa and functions were correlated with clinical parameters of gout patients. PERMANOVA based on the microbial gene-level profile revealed that disease status (gout versus healthy) was the strongest factor (in terms of both *P* values and *R*^2^ in PERMANOVA; Supplementary Data 1), followed by SCr, SUA, and CRP (FDR *P* < 0.05).

**Fig. 3:**
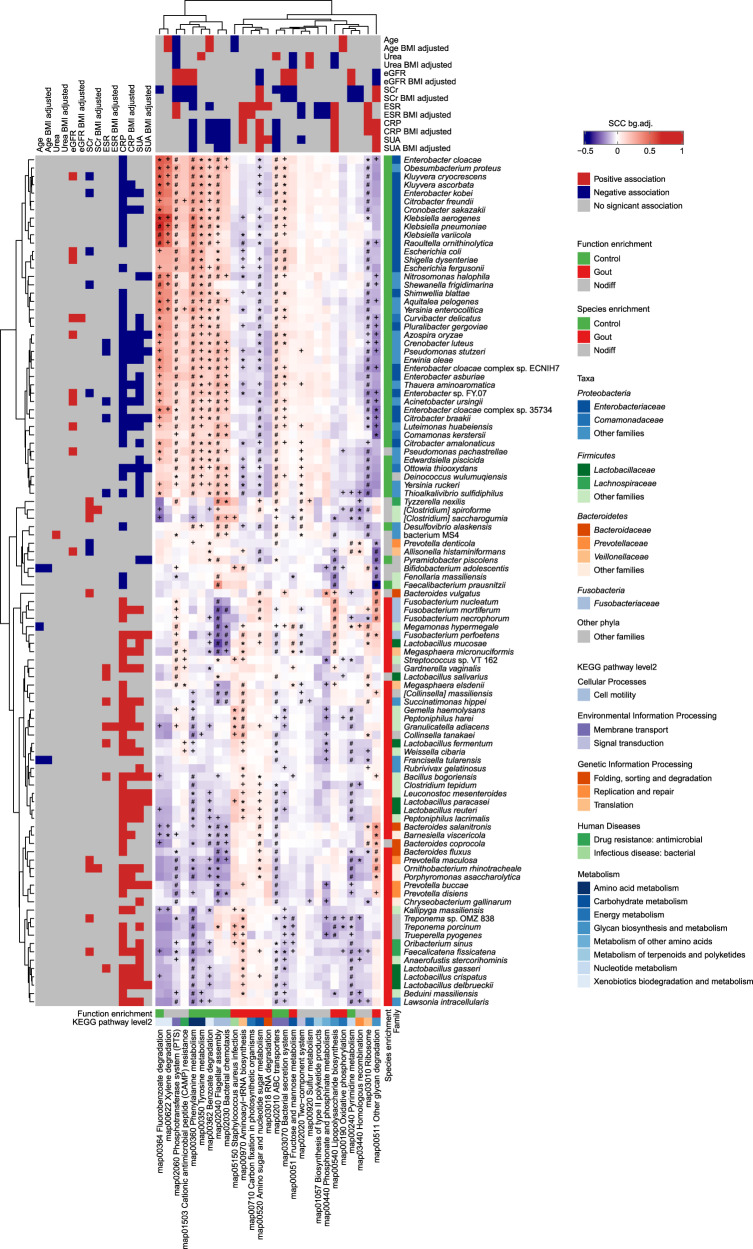
The three-pronged association heatmap of bacterial species, KEGG pathways and clinical parameters. The left panel denotes the significant Spearman correlations (FDR *P* < 0.05) between bacterial species and clinical indices with or without adjustment for BMI, colored by positive (red), negative (blue), or nonsignificant correlation (gray). The top panel denotes the significant association (Wilcoxon rank-sum test, FDR *P* < 0.05) between KEGG pathways and clinical indices with or without adjustment for BMI. The bottom panel denotes the functional category and directionality of enrichment in gout and controls for KEGG pathways colored by healthy control-enriched (green), gout patient-enriched (red) or no significant difference (gray). The right panel denotes the family-level taxonomy and directionality of enrichment in gout and controls for species colored by healthy control-enriched (green), gout patient-enriched (red), or no significant difference (gray). The centered heatmap denotes the median Spearman correlation coefficient between each species and all KOs within a given KEGG pathway, adjusted for background distribution by subtracting the median Spearman correlation coefficient between the species and all other KOs outside the pathway (red: positive correlation; purple: negative correlation). A Wilcoxon rank-sum test was performed between Spearman correlation coefficients between each species and all KOs within a given KEGG pathway and Spearman correlation coefficients between the species all other KOs outside the pathway. Wilcoxon rank-sum test: ‘plus’ denotes FDR *P* < 0.05; ‘asterisk’ denotes FDR *P* < 0.01, ‘hash’ denotes FDR *P* < 0.001.

We performed a three-pronged correlation analysis between microbial species, functions and clinical parameters (Fig. 3). Consistent with PERMANOVA, SUA and CRP exhibited strongest correlations with microbial species and functional pathways. SUA was positively correlated with 25 gout-enriched species, whereas it was negatively correlated with 22 control-enriched species, 20 of which belonged to *Proteobacteria*. CRP exhibited significant positive correlations with 40 gout-enriched species and negative correlations with 38 control-enriched species. The *Proteobacteria* species, in particular those from *Enterobacteriaceae*, showed positive correlations with pathways in amino acid metabolism, benzoate degradation and cell motility (i.e. flagella assembly and chemotaxis), which together exhibited inverse correlations with SUA and CRP. Conversely, the gout-enriched species from *Firmicutes* and *Bacteroidetes* exhibited moderate correlations with gout-enriched functional pathways such as carbohydrate metabolism, energy metabolism and lipid A biosynthesis. These results suggested that the decreased abundances of *Enterobacteriaceae* species likely contributed to reduced functional potentials in amino acid metabolism and environmental sensing, which together resulted in increased uric acid and systemic inflammation in gout patients. The depletion of microbial genes in flagellar assembly and bacterial chemotaxis was consistent with previous findings in AS^8^ and type 2 diabetes (T2D)^37^. The correlation patterns mostly persisted when controlled for BMI. Two renal function indicators, SCr and eGFR, were associated with 9 and 8 species, respectively. Few species were associated with ESR, urea and age.

### A classifier using gut metagenomic genes for gout

**Fig. 4:**
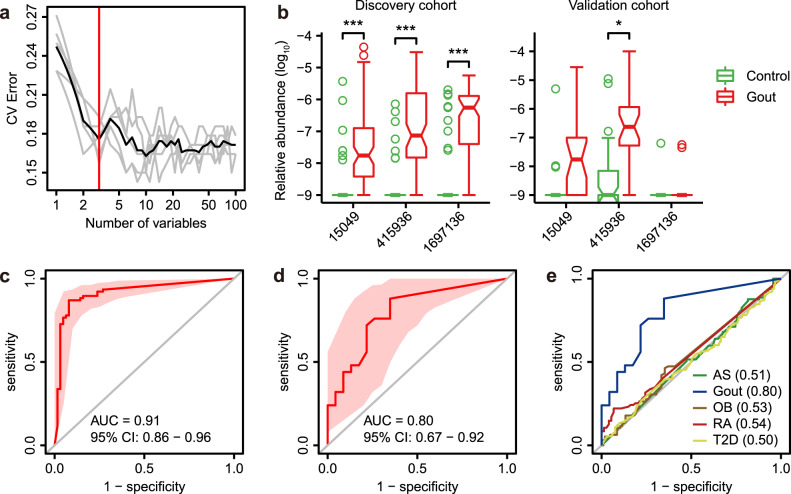
The gut metagenomic classifier for gout. **a** The model was trained using relative abundance of microbial genes in discovery cohort. All microbial genes were first ranked based on their variable importance and then added sequentially into the model. The error curves were plotted for the five trials of 10-fold cross-validation in random forest classification as the number of genes increased. The black curve indicates the average cross-validation error of the five trials (in gray). The minimum error in the averaged curve plus the standard deviation at that point was used as the cutoff for feature selection. The model containing the smallest number of genes with an error below that cutoff was chosen as the optimal classifier. The red line marks the number of genes in the optimized model. **b** The relative abundance of three microbial gene markers in discovery and validation cohorts. Wilcoxon rank-sum test: ‘asterisk’ denotes FDR *P* < 0.05; ‘double asterisks’ denote FDR *P* < 0.01; ‘triple asterisks’ denote FDR *P* < 0.001. **c** Receiver operating curve (ROC) for the discovery samples. **d** ROC for the validation samples (healthy control, *n* = 23; gout patient, *n* = 25). **e** ROCs for gout and four public case-control metagenomic datasets for ankylosing spondylitis (AS), obesity (OB), rheumatic arthritis (RA), and type 2 diabetes (T2D) using three gout-associated gene markers. The AUC for each disease was shown in parenthesis. For all box and whisker plots, the center line represents median. The bounds of box represent the first and third quartiles. The upper whisker extends from the hinge to the largest value no further than 1.5 * interquartile range (IQR) from the hinge. The lower whisker extends from the hinge to the smallest value at most 1.5 * IQR of the hinge. The notch represents a confidence interval around the median as the median ± 1.58*IQR/sqrt(*n*).

The distinct microbial differences between gout patients and healthy controls prompted us to investigate if the gut microbiome had the potential to discriminate gout patients from healthy controls. A disease classifier was constructed based on the microbial gene-level profile using a random forest model in the discovery cohort. The top 100 differentially abundant genes were included in the disease classifier. After feature selection based on 10-fold cross-validation (see methods), three genes (gene ID: 15049 [exo-alpha-sialidase], 415936 [N-6 DNA methylase], 1697136 [relaxase/mobilization nuclease domain-containing protein]) were retained with optimal performance, based on which a classification model was established (Fig. 4a and Supplementary Data 1). All three genes were significantly enriched in gout patients in discovery cohort (Fig. 4b, FDR *P* < 3.76e–7) and two of them (15049 and 415936) showed increasing trend in validation cohort (FDR < 0.09). The area under the receiver operating curve (AUC) reached 0.91 and 0.80 for discovery and validation cohorts, respectively (Fig. 4c, d and Supplementary Data 1). To assess gout specificity for these markers, we retrieved public case-control metagenomic sequencing data for AS (*n* = 211), RA (*n* = 169), T2D (*n* = 268) and obesity (OB, *n* = 200) (see methods), processed these datasets using the same pipeline and assessed the discriminating potential of the three genes between patients and controls. The AUCs in these cohorts were ranged between 0.50–0.54, suggesting the three-gene signature was gout-specific (Fig. 4e). The three genes were taxonomically annotated to *F. mortiferum* (species-level), *Bacteroides* (genus-level) and *Bacteroides* (genus-level) respectively. *F. mortiferum* was significantly elevated in gout (FDR *P* = 5.53e–4, Supplementary Data 1) and all differential *Bacteroides* spp. were also gout-enriched, supporting possible relevance of the gene markers in gout biology. In comparison, feature selection identified 13 species-level markers for gout, which had reduced AUCs of 0.86 and 0.63 for discovery and validation cohorts, respectively (Supplementary Fig. 10). These results suggest that gout-specific microbial genes have the potential as diagnostic markers for the disease.

### Effect of uric-acid-lowering and anti-inflammatory interventions on gut metagenome in gout

**Fig. 5:**
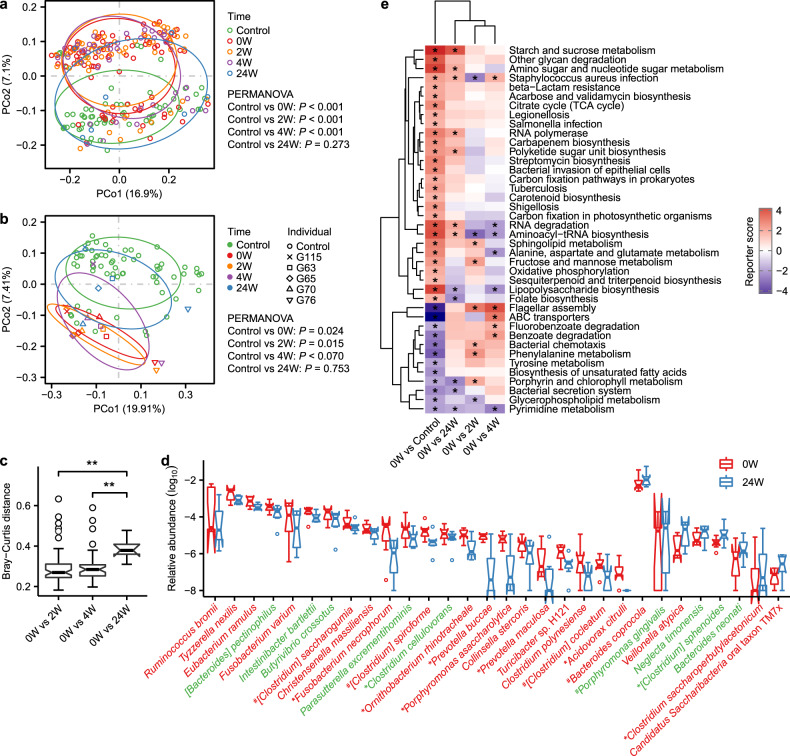
Alternation of gut microbiota by therapeutic intervention in gout. **a** PCoA based on Bray–Curtis distance at gene level of healthy controls and gout patients before and after treatment (0W, *n* = 77; 2W, *n* = 61; 4W, *n* = 38; 24W, *n* = 7). The 95% confidence ellipses were shown for all subgroups. **b** PCoA based on Bray–Curtis distance at gene level of healthy controls and five time point paired gout patients. The 95% confidence ellipses were shown for all subgroups. **c** Box and whisker plot of beta diversity of gout patients between before and after treatment. Wilcoxon rank-sum test: Double asterisks denote *P* < 0.01. **d** The relative abundance of bacterial species was modulated after 24 weeks treatment (*P* < 0.05, paired Wilcoxon rank-sum test, *n* = 7). Bacterial species in color green and red indicate healthy control-enriched and gout patient-enriched in discovery cohort, respectively. **e** Microbial gene functions were changed after treatment. Purple, enriched in healthy controls or patients after treatment; red, enriched in patients before treatment. Asterisk denotes reporter score of pathways > 1.65 or < −1.65. For all box and whisker plots, the center line represents median. The bounds of box represent the first and third quartiles. The upper whisker extends from the hinge to the largest value no further than 1.5 * interquartile range (IQR) from the hinge. The lower whisker extends from the hinge to the smallest value at most 1.5 * IQR of the hinge. The notch represents a confidence interval around the median as the median ± 1.58*IQR/sqrt(*n*).

Previous studies have demonstrated the influence of therapeutic drugs on gut microbiota^38^. We assessed the gut microbiome dynamics for a subset of gout patients receiving therapeutic intervention at 2-week (2W, *n* = 61), 4-week (4W, *n* = 38), and 24-week (24W, *n* = 7) post-baseline time points. Most patients received uric-acid-lowering and anti-inflammatory drugs (Supplementary Fig. 11a and Supplementary Data 1). We excluded 9, 2, and 2 fecal samples from 2W, 4W, and 24W groups, respectively, due to nonconventional medication use. The microbial gene number and Shannon index were not significantly altered in gout patients over the period of drug intervention (Supplementary Fig. 11b, c). However, principal coordinate analysis (PCoA) showed the samples at 24W were separated from the rest of patient samples and closer to healthy controls (Fig. 5a). Likewise, samples at 24W had significantly higher Bray–Curtis dissimilarity from the baseline samples compared to those at 2W or 4W (0W–2W vs 0W–24W, *P* = 0.0023; 0W–4W vs 0W–24W, *P* = 0.0017; Fig. 5c), indicating a greater impact by intervention on the gut microbiome at 24 weeks. Considering that the unmatched sample size at different time points may bias the results, we selected five gout patients whose fecal samples were collected at all four time points and the results were consistent with those using all samples (Fig. 5b; Supplementary Fig. 11d, e). Compared with baseline, the abundance of 22 bacterial species, including 9 gout-enriched species, were decreased at 24W, whereas 8 species, including 2 control-enriched species, were increased (Fig. 5d). Functionally, 10 KEGG pathways that were significantly altered in gout patients versus controls at baseline showed significant reversing trends at 24W (Fig. 5e). There results suggested that uric-acid-lowering and anti-inflammatory drugs may partially restore the gut microbes at 24-week treatment. The medications for gout, including benzbromarone, allopurinol, colchicine, celecoxib, and etoricoxib, have been shown to impact gut bacterial growth in vitro by single drug inhibition experiment^39^. Considering the drugs interaction for most gout patients receiving both uric-acid-lowering and anti-inflammatory drugs in this study, there may be a more sophisticated relationship in vivo between these drugs and gut microbiota. Due to the observatory nature of our study, the impact of specific drugs on the gut microbiome needs to be further studied in interventional clinical trials of larger cohorts.

### Comparison of gut metagenome between gout and other autoimmune and metabolic disorders

**Fig. 6:**
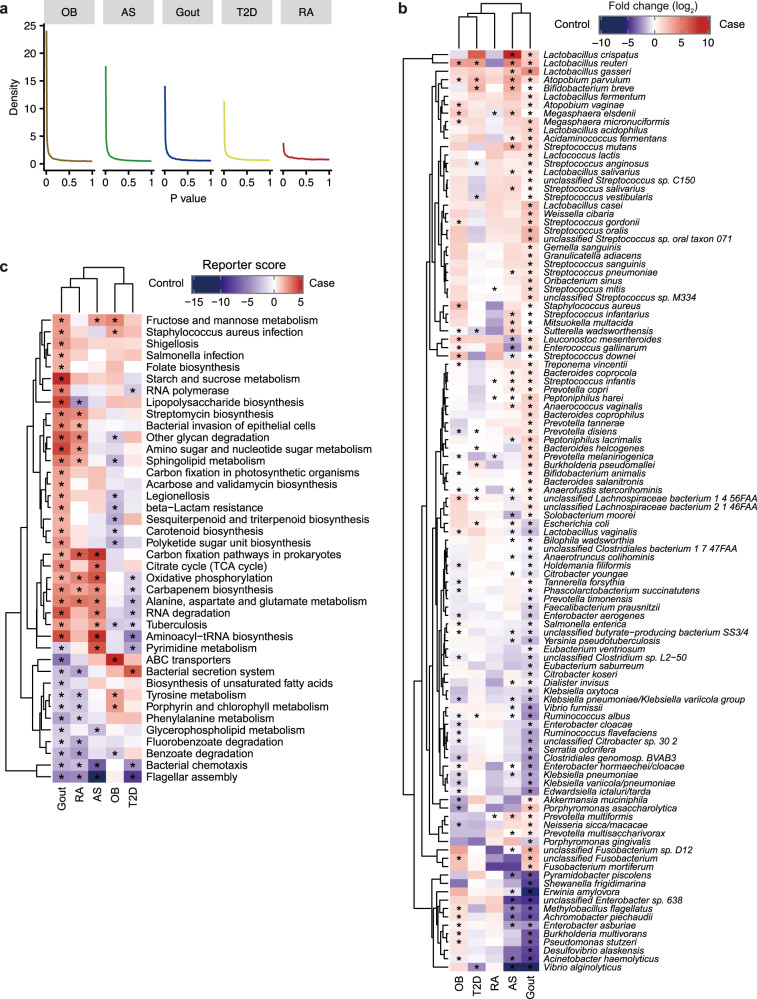
Comparison of gut microbiome between gout and other autoimmune and metabolic diseases. **a** The distribution of *P* values by Wilcoxon rank-sum test for all microbial genes in case-control comparison within each of the AS (*n* = 211), gout (*n* = 140), OB (*n* = 200), RA (*n* = 169), and T2D (*n* = 268) datasets. **b** Comparison of differential species in AS, gout, OB, RA and T2D. Purple, enriched in healthy controls; red, enriched in patients; Wilcoxon rank-sum test: asterisk denotes FDR *P* < 0.05. **c** Comparison of microbial gene functions in AS, gout, OB, RA and T2D. Purple, enriched in healthy controls; red, enriched in patients. Asterisk denotes reporter score of pathways > 1.65 or < −1.65.

To better understand the role of gut microbes in gout in the context of broader chronic disorders, we compared the gut microbial signature of gout with those of AS, RA, T2D, and OB using public case-control metagenomic datasets, of which the former two are autoimmune diseases, and the latter two are metabolic diseases. The *P* value distribution of all genes in case-control comparison within each dataset suggested that the degree of overall gut microbial dysbiosis was intermediate in gout patients compared with the other four diseases (Wilcoxon rank-sum test, Fig. 6a). Similar results were observed when controlling for the study sample size (Supplementary Fig. 12). The differential bacterial species and gene families, as obtained in case-control comparison within each dataset, were then cross-compared between studies, to assess microbial taxonomic and functional signatures for each type of disease. For taxonomic signature, gout was most similar to AS patients as they shared 40 differential species (Fig. 6b and Supplementary Fig. 13a), whereas RA was clustered with metabolic diseases OB and T2D. For microbial functions, gout was clustered with both RA and AS (Fig. 6c and Supplementary Fig. 13b). Specifically, genes in oxidative phosphorylation, alanine, aspartate and glutamate metabolism, carbon fixation pathways in prokaryotes and carbapenem biosynthesis were commonly enriched in gout, AS and RA patients, whereas genes in bacterial chemotaxis and flagellar assembly were depleted in all three disorders. These results provide evidence that the dysbiosis signature of gout is likely more similar to those in autoimmune than metabolic diseases, which implies that gut microbiota may exert common influence on the development of autoimmune diseases. However, how gut microbiota interact with host in autoimmune diseases remains unclear and warrants investigation in mechanistic studies.

## Conclusion

In summary, we identified taxonomic and functional signatures in the gut microbiome associated with gout, and proposed a hypothetical model of how gut microbes may influence the development of gout based on our results (Supplementary Fig. 14). The enrichment of species in *Bacteroides* and *Prevotella* over those in *Enterobacteriaceae* in gout may contribute to an altered biosynthesis of six-acyl-chain lipid A to those with four or five acyl chains, and together could exhibit negative effects in host immune stimulation and endotoxin tolerance. Meanwhile, depletion of *Enterobacteriaceae* species may contribute to dysfunction in uric acid degradation which lead to increased systemic uric acid accumulation and inflammations in gout. Other microbial functions such as SCFA production and flagellar assembly may help maintain a healthy gut microenvironment and their depletion in gout may result in increased local and systemic inflammations through modifying their host receptors. Our results showed a dysbiosis of gut microbiome in gout that was associated with increased SUA and systemic inflammation and may be partially restored by uric-acid-lowering and anti-inflammatory drug interventions over time. Future multi-omic studies on larger longitudinal cohort, together with animal model experiments, are needed to validate our findings toward a better understanding on the underlying mechanisms of gut microbiota in gout.

## Methods

### Subjects and sample collection

This study was approved by the Medical Ethics Committee of the Second Affiliated Hospital of Guangzhou University of Chinese Medicine (B2016-103-01). All participants provided written informed consent. Patients were diagnosed with gout as determined by the 2015 ACR/EULAR classification criteria^40^ and suitability for the treatment in this study. Patients and healthy controls had to meet the following criteria: (1) 15–69 years of age; (2) no antibiotics and glucocorticoid use within 3 months and 1 month, respectively; (3) no gastrointestinal diseases, such as gastrointestinal surgery, Crohn’s disease, ulcerative colitis, or acute diarrhea; (4) no history of severe, progressive or uncontrolled cardiac, hepatic, renal, mental, or hematological disease; and (5) no history of drug abuse. All participants were informed about the purpose of this study and provided written informed consent.

Between May 2016 and September 2018, we recruited 102 male acute gout patients (SUA, 543.1 ± 128.4 μmol/L) and 86 age-matched male healthy controls (SUA, 358.6 ± 52.3 μmol/L) for this study. 140 subjects were recruited from 2016 to 2017 and used as discovery cohort. An additional 48 subjects were recruited in 2018 as validation cohort. For discovery cohort, after collecting fecal samples at baseline, gout patients were treated with uric-acid-lowering (benzbromarone, allopurinol, febuxostat) and anti-inflammatory drugs (colchicine, celecoxib, etoricoxib, betamethasone, voltaren), and fecal samples were collected after drugs treatment for 2 weeks (2W, *n* = 70), 4 weeks (4W, *n* = 40), and 24 weeks (24W, *n* = 9). A total of 307 fecal samples were collected and frozen at −80 °C. Food frequency questionnaire was collected from all participants to assess dietary differences between two groups and showed no significant difference on specific dietary habits, including alcohol drinking, probiotics/prebiotics and completely vegetable-based diet (Supplementary Data 1).

Blood samples were collected and frozen at −80 °C until analysis. SUA, SCr, urea nitrogen, CRP, and estimated glomerular filtration rate (eGFR) were measured using the Cobas 8000 modular analyzer (Roche, Switzerland), and ESR was measured using the Test-1 analyzer (Alifax, Italy).

### DNA extraction and library construction

The collected fecal specimens were centrifuged at 12000 × *g* at room temperature for 5 min and the supernatant was discarded. 200 mg pellet was weighted from each sample and used for total bacterial DNA extraction with the E.Z.N.A Stool DNA Kit (OMEGA Bio-tek, USA) according the manufacturer’s instructions. The quality of DNA was analyzed using Qubit (Invitrogen, USA) and 1% agarose gel electrophoresis. The detail of DNA library construction was described in Supplementary File 1. The final DNA library was determined the average insert size using the Agilent 2100 Bioanalyzer (Agilent Technologies, USA) and quantified by ABI StepOnePlus Real-Time PCR system (Applied Biosystems, USA).

### Metagenomic sequencing

Paired-end metagenomic sequencing was performed on the Illumina HiSeq 4000 platform with an insert size of 350 bp and paired-end (PE) reads of 150 bp for each sample. After removing adaptors, low quality and ambiguous bases from the raw reads, the remaining reads were aligned to human genome reference (hg19) by SOAPaligner (v2.22, parameters: -m 280 -x 420 -r 1 -l 32 -s 75 -c 0.9) to remove human host DNA contamination. The average rate of host contamination was 0.52 ± 2.06%. Finally, 2768.76 Gb of high-quality PE reads for the 307 samples were acquired with an average of 9.02 Gb per sample (Supplementary Data 1).

### Construction of gene, phylum, genus, species and KO profiles

The clean reads were aligned to the 11,446,577 genes in the reference gene catalog^41^ using SOAPaligner (v2.22, parameters: -m 200 -x 1000 -r 2 -v 13 -l 32 -s 75 -c 0.95), and 72.79 ± 2.89% reads (*n* = 307) were mapped. The gene abundance profile was calculated according to Wen et al.^8^, with minor modifications. Specifically, two types of alignments were considered for calculation of gene abundance: (1) both pairs of reads matched to the same genes with the correct insert-size; (2) one of the paired-end reads matched to the end of the genes, while the other was located outside of the gene. The matched reads were then split into two parts: (1) *U*: reads match this gene only; and (2) *M*: reads also match another gene. The gene abundance was also split into two parts, Ab(U) and Ab(M). The unique part Ab(U) was calculated as the number of reads divided by the length of the gene. For the multiple part Ab(M), each of the reads in set *M* was assigned to several parts according to the unique abundance of genes with which the reads matched. For a given sample, the relative abundance of gene *i* was calculated using below procedure.

Step 1: Calculation of the abundance of uniquely matched reads (Ab(U)):

1 $${{{\mathrm{Ab}}}}({{{\mathrm{U}}}}) = \frac{{{{\mathrm{U}}}}}{{{{\mathrm{L}}}}}$$

Step 2: Calculation of the abundance of multiple matched reads (Ab(M)):

2 $${{{\mathrm{Co}}}} = \frac{{{{{\mathrm{Ab}}}}({{{\mathrm{U}}}})}}{{\mathop {\sum }\nolimits_{{{{\mathrm{i}}}} = 1}^{{{\mathrm{N}}}} {{{\mathrm{Ab}}}}({{{\mathrm{U}}}}_{{{\mathrm{i}}}})}}$$

3 $${{{\mathrm{Ab}}}}({{{\mathrm{M}}}}) = \frac{{{{{\mathrm{M}}}} \ast {{{\mathrm{Co}}}}}}{{{{\mathrm{L}}}}}$$

Step 3: Calculation of the relative abundance of gene (Ab(G)):

4 $${{{\mathrm{Ab}}}}({{{\mathrm{G}}}}) = \frac{{{{{\mathrm{Ab}}}}({{{\mathrm{U}}}}) + {{{\mathrm{Ab}}}}({{{\mathrm{M}}}})}}{{\mathop {\sum }\nolimits_{{{\mathrm{j}}}} ({{{\mathrm{Ab}}}}({{{\mathrm{U}}}}) + {{{\mathrm{Ab}}}}({{{\mathrm{M}}}}))}}$$

*U*: The number of reads that were uniquely matched to gene ${i}$.

*L*: The length of gene ${i}$.

*M*: The number of reads that were non-uniquely matched to gene ${i}$.

*N*: The number of different genes to which the read in M was aligned.

After removing genes detected in less than 10% of the discovery samples (*n* = 259), 1,564,977 genes remained. To improve the gene taxonomy annotation, genes were aligned to the National Center for Biotechnology Information (NCBI) microbial reference genomes (including 5847 microbial genomes, v20171114) and NT database (v20170924) using BLAT (v.36) and Megablast (v2.2.26) with default parameters, respectively. The alignments of each gene with at least 70% gene length coverage and 65% identity were retained. Each gene was assigned the taxonomy of the alignment(s) with 50% or higher consensus above the similarity threshold for taxonomic rank (>65% for phylum, >85% for genus and >95% for species), according to the scheme in Li et al.^42^. The relative abundances of phyla, genera, species and KOs were calculated by aggregating the relative abundance of the genes that were assigned to the corresponding taxonomic or functional ranks. MetaPhlAn3 was used for reads-based taxonomic profiling to complement with the above results^24^.

### Rarefaction curve, gene counts and biodiversity analysis

Rarefaction analysis was performed to assess the gene richness in the healthy controls and gout patients. For a given number of samples, we performed random sampling 100 times in the cohort with replacement and estimated the total number of genes that could be identified from these samples by the Chao2 richness estimator^43^. The total gene counts in each sample were calculated as the number of genes in reference gene catalog that were mapped by the reads^44^. The alpha diversity and beta diversity were estimated by the Shannon index and Bray–Curtis distance, respectively. To adjust for the effect of the various data sizes among the samples, these analyses were based on the gene profile that was randomly sampled to 11 million matched reads.

### Gene function analysis

Differentially enriched KEGG pathways and modules were identified according to reporter score^45^ from the *Z*-scores of individual KOs (KEGG database release 79). Pathways or modules were considered significantly different if the |reporter score| > 1.65, corresponding to 95% confidence to a normal distribution.

For analysis of urate degradation genes, species that contain at least one urate degradation gene in the KEGG database (v79) were collected, and the corresponding 16S sequences were downloaded from the Ribosomal Database Project (set14). Then, 16S sequences were aligned by PyNAST (v1.2.2), and trees were constructed by fasttree (v2.1.7). The tree and heat map were visualized in iTOL^46^.

The protein sequences of SCFA-producing enzymes were obtained from the NCBI database^47^. Genes in the reference gut microbiome gene catalog were aligned to these sequences using BLASTP (v2.2.26, best hit with *e*-value < 1e−5, identity > 70% and coverage > 70%), and their relative abundance were calculated as KOs.

### Random forest classifier

We constructed a classifier to discriminate samples of healthy controls and gout patients based on a random forest model (randomForest 4.6–14 package) using the relative abundance of microbial genes^18^. A 10-fold cross-validation approach was employed with five trials to evaluate the performance of the prediction model. Feature selection was performed according to the scheme in Feng et al.^18^. Specifically, all microbial genes were ranked based on their variable importance and added sequentially into the model. The averaged cross-validation errors were plotted as the number of genes increased. The minimum error in the averaged curve plus the standard deviation at that point was used as the cutoff for feature selection. All gene sets with an error less than the cutoff were listed and the set with the smallest number of genes was chosen as the optimal set. The receiver operating characteristic curves (ROC) for both discovery and validation cohorts were plotted and the area under curve (AUC) was calculated using the pROC package^48^.

### Comparison of the gut microbiota between gout and other diseases

Published metagenomic sequencing data of AS (SRP100575 and ERP005860; 114 healthy controls and 97 patients with AS)^8^, RA (ERP006678; 74 healthy controls and 95 patients with RA)^10^, type 2 diabetes (T2D) (SRP008047 and SRP011011; 185 healthy controls and 183 patients with T2D)^37^ and obesity (OB) (ERP013562; 105 healthy controls and 95 patients with OB)^49^ was retrieved from NCBI or the European Bioinformatics Institute (EBI). All sequencing reads were mapped to the same reference gut gene catalog and analyzed for gene function using the same pipeline as the gout cohort.

### Statistical analysis

All statistical analyses were performed by R (v3.4.0). Differential relative abundance of genes, taxa and KOs were detected by Wilcoxon rank-sum test with an adjusted *P* value (corrected by the Benjamini–Hochberg) < 0.05. Enrichment in healthy controls or gout patients was determined according to the higher mean rank-sum. The enterotype of each sample in discovery cohort was identified by the PAM clustering algorithm using the relative abundance of genus. Calinski–Harabaze (CH) index was used to assess the optimal number of clusters^50^. Permutation multivariate analysis of variance (PERMANOVA) based on Bray–Curtis distance was performed at the gene level to assess the impact of the clinical indices on gut microbiota, and the permutations was set to 9999. The correlations between the relative abundance of differentially genera and KOs involved to urate degradation, the relative abundance of species and clinical indices were calculated by Spearman’s rank correlation coefficient and visualized by heatmap in R using the ‘complexheatmap’ package. The association between species, BMI and other confounders was calculated by MaAsLin with default parameters^51^.

Associations between bacterial species, bacterial functions and clinical indices were characterized according to the method in Pedersen et al.^52^. Specifically, to assess association between each species and each microbial KEGG pathway, a Spearman correlation analysis was performed between the abundances of the species and each individual KO in the KEGG pathway, and a Wilcoxon rank-sum test was then performed between the Spearman correlation coefficients for all KOs within the pathway and all other KOs outside the pathway (the so-called ‘background distribution’). The strength of correlation between the species and the KEGG pathway was denoted as the median Spearman correlation coefficient between the species and all KOs within the pathway, corrected for background distribution by subtracting the median Spearman correlation coefficient between the species and all other KOs outside the pathway. The association between KEGG pathways and clinical indices was performed in a similar manner. The association between bacterial species and clinical indices was performed by standard Spearman correlation. Additional partial Spearman correlation analyses were performed to adjust for BMI in associating with clinical indices.

### Reporting summary

Further information on research design is available in the Nature Research Reporting Summary linked to this article.

## Supplementary information

Supplementary Information

Supplementary Data 1

Reporting Summary

## Data Availability

The metagenomic shotgun sequencing data for all samples have been deposited in the CNGB Nucleotide Sequence Archive (CNSA) under accession code CNP0000284. Other data that support the findings of this study are available within the paper and its Supplementary Information files or from the corresponding author upon reasonable request.

## Code availability

The key computer codes for the analyses in this study are available in GitHub (https://github.com/hyf-2021/Gout-script).
