# Supplementary material for: Metagenomic analysis revealed the potential role of gut microbiome in gout
Source: NPJ Biofilms Microbiomes. 2021 Aug 9;7:66. doi: 10.1038/s41522-021-00235-2 (PMC8352958; doi:10.1038/s41522-021-00235-2)
Supplement: Supplementary file 1 — Supplementary Information [file 41522_2021_235_MOESM1_ESM.pdf]

Metagenomic analysis revealed the potential role of gut microbiome in gout

Supplementary document

Supplementary Methods

DNA library construction

1.5 μg DNA was fragmented by Covaris E210 (Covaris, USA). The fragmented DNAs were tested by Gel-Electrophotometric. The fragmented DNAs were combined with End Repair Mix, incubated at 20 °C for 30 min. The end-repaired DNA was purified with QIAquick PCR Purification Kit (QIAGEN, Germany), then A-Tailing Mix was added and incubated at 37 °C for 30 min. The purified Adenylate 3’-Ends DNA was combined Adapter and Ligation Mix, incubated the ligation reaction at 20 °C for 15 min. Adapter-ligated DNA was selected by running a 2% agarose gel to recover the target fragments. The gel was purified with QIAquick Gel Extraction Kit (QIAGEN, Germany). Several rounds of PCR amplification with PCR Primer Cocktail and PCR Master Mix were performed to enrich the Adapter-ligated DNA fragments. Then the PCR products were selected by running 2% agarose gel to recover the target fragments. The gel was purified with QIAquick Gel Extraction Kit (QIAGEN, Germany).

Supplementary Data 1. The supplementary data 1 contains below information as data tables.

Supplementary Table 1. Phenotype of all participants.

Supplementary Table 2. Alterations in clinical characteristics after drug treatment in gout patients.

Supplementary Table 3. Gout associated phyla.

Supplementary Table 4. Gout associated genera.

Supplementary Table 5. Gout associated species.

Supplementary Table 6. Gout associated taxa detected by MetaPhlAn3.

Supplementary Table 7. Gout associated KOs.

Supplementary Table 8. Gout associated pathways and modules.

Supplementary Table 9. Spearman's rank correlation between Urate degradation-associated KOs and genera.

Supplementary Table 10. Urate degradation-associated species in KEGG database.

Supplementary Table 11. PERMANOVA for the influence of gout. Bray Curtis distance and 9,999 permutations.

Supplementary Table 12. The 3 gene markers was identified to discriminate healthy controls and gout patients base on a random forest model.

Supplementary Table 13. The probability of gout was predicted in discovery samples and validation cohort according to the 3 microbial gene biomarkers selected by random forest model.

Supplementary Table 14. Dietary pattern of the discovery cohort.

Supplementary Table 15. Data production, quality control and IGC database alignment.

Supplementary figures

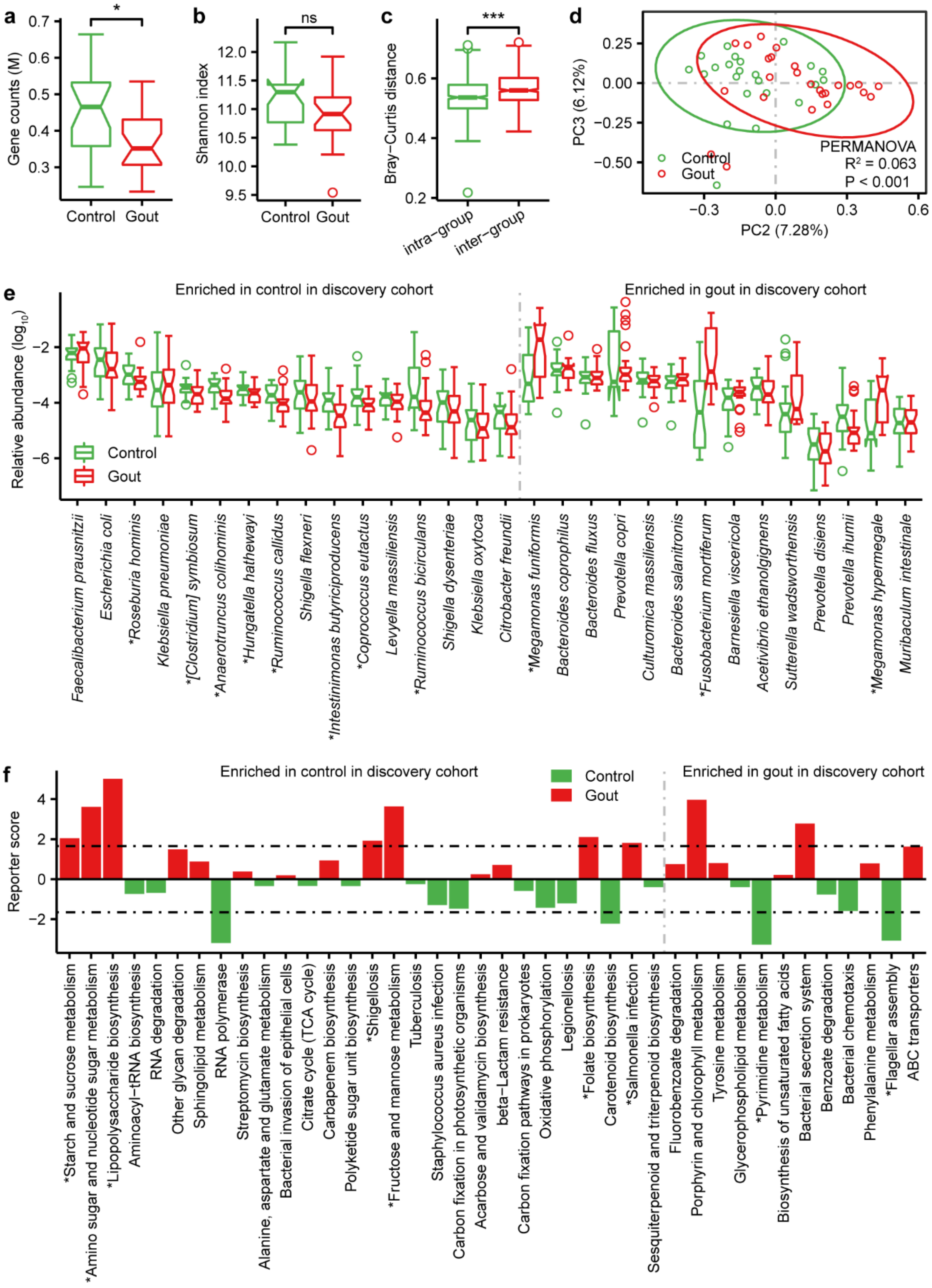

Supplementary Figure 1. The alpha and beta diversity, differential species and pathways in validation cohort. **(a)** Box and whisker plot of gene count in the healthy controls and gout patients. **(b, c)** Box and whisker plots of alpha diversity (Shannon index) and beta diversity (Bray-Curtis distance) at the gene level. Wilcoxon rank-sum test was used to determine

significance. ‘*’ denotes $P < 0.05$; ‘**’ denotes $P < 0.01$; ns denotes no significant. **(d)** Principal component analysis (PCA) based on the gene abundance profile. **(e)** The relative abundance of bacterial species which showed in Figure 1i in validation cohort. ‘*’ denotes species with $P < 0.05$ in both discovery and validation cohort. **(f)** Reporter score of pathways in validation cohort. ‘*’ denotes pathways with |reporter score| > 1.65 both in discovery and validation cohort. For all box and whisker plots, the center line represents median. The bounds of box represent the first and third quartiles. The upper whisker extends from the hinge to the largest value no further than 1.5 * interquartile range (IQR) from the hinge. The lower whisker extends from the hinge to the smallest value at most 1.5 * IQR of the hinge. The notch represents a confidence interval around the median as the median +/- 1.58*IQR/sqrt(n).

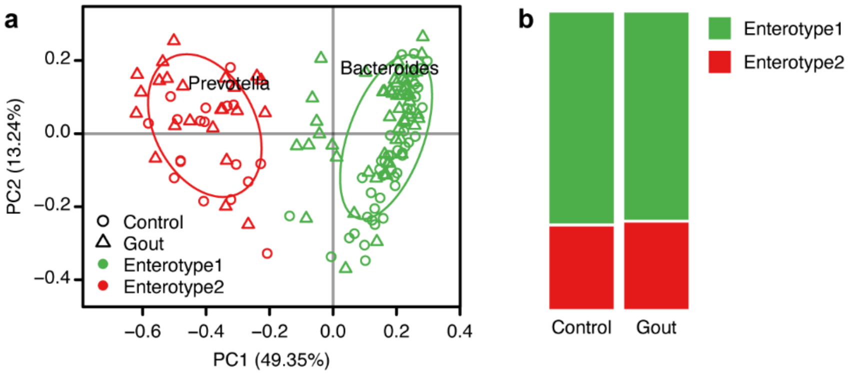

Supplementary Figure 2. Identification of gut enterotypes in gout patients and healthy controls. **(a)** Two enterotypes were identified by PCA at the genus level. Predominant genera in enterotype1 and enterotype2 were *Bacteroides* and *Prevotella*, respectively. **(b)** Distribution of enterotypes in gout patients and healthy controls. $P = 0.8541$, fisher's exact test.

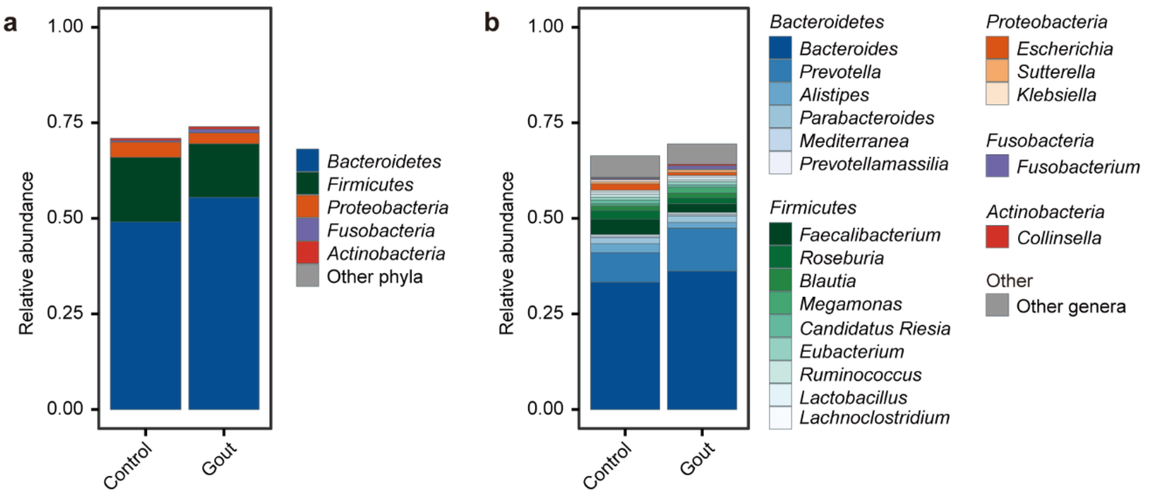

Supplementary Figure 3. Gut microbial composition of gout patients and healthy controls. The relative abundance of major phyla **(a)** and genera **(b)** were shown and the unclassified taxa were not included.

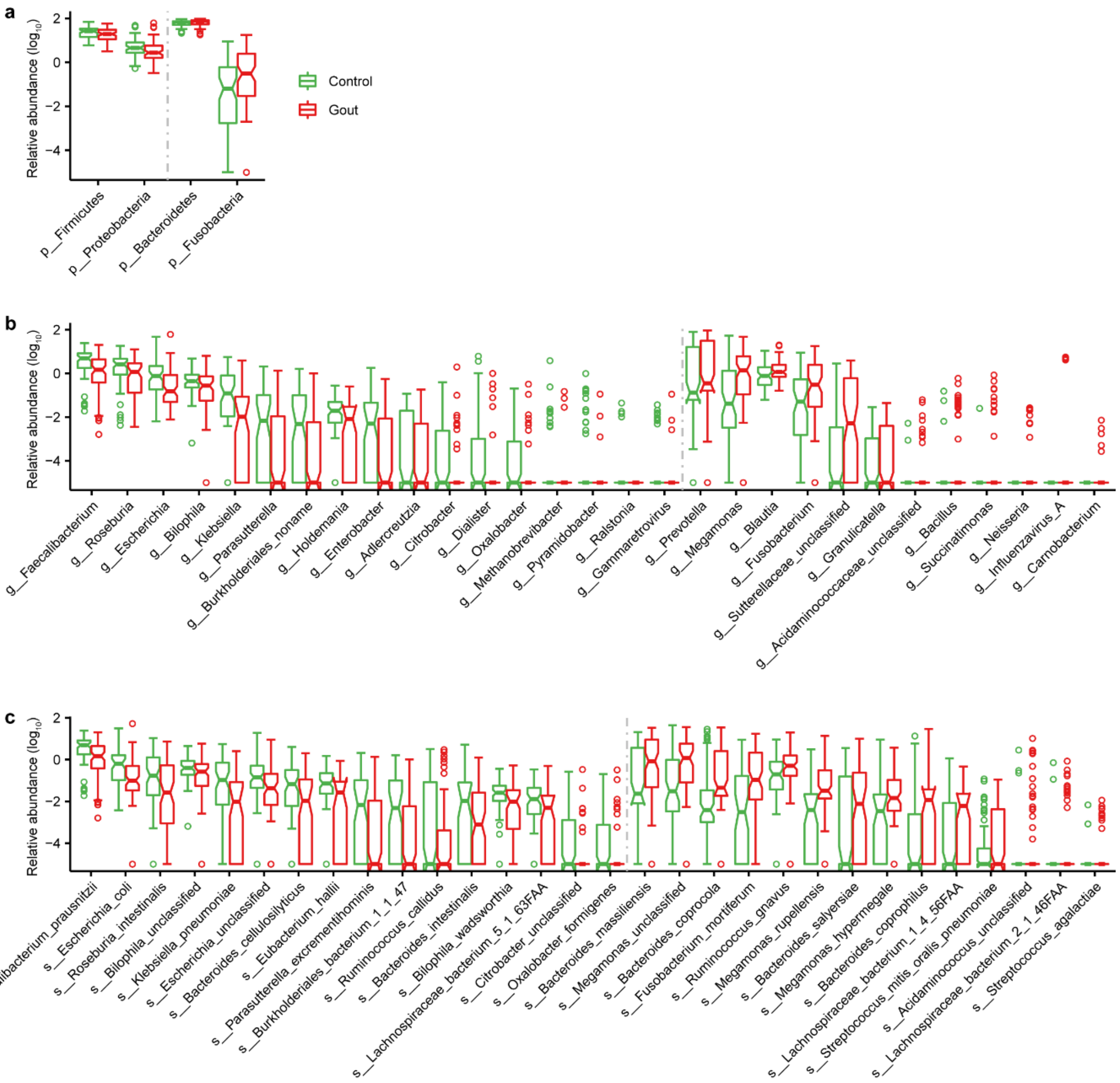

Supplementary Figure 4. The top 4, 30 and 30 differentially abundant **(a)** phylum-, **(b)** genus- and **(c)** species-level taxa between gout and controls as obtained using MetaPhlAn3 analysis. For all box and whisker plots, the center line represents median. The bounds of box represent the first and third quartiles. The upper whisker extends from the hinge to the largest value no further than 1.5 * interquartile range (IQR) from the hinge. The lower whisker extends from the hinge to the smallest value at most 1.5 * IQR of the hinge. The notch represents a confidence interval around the median as the median +/- 1.58*IQR/sqrt(n).

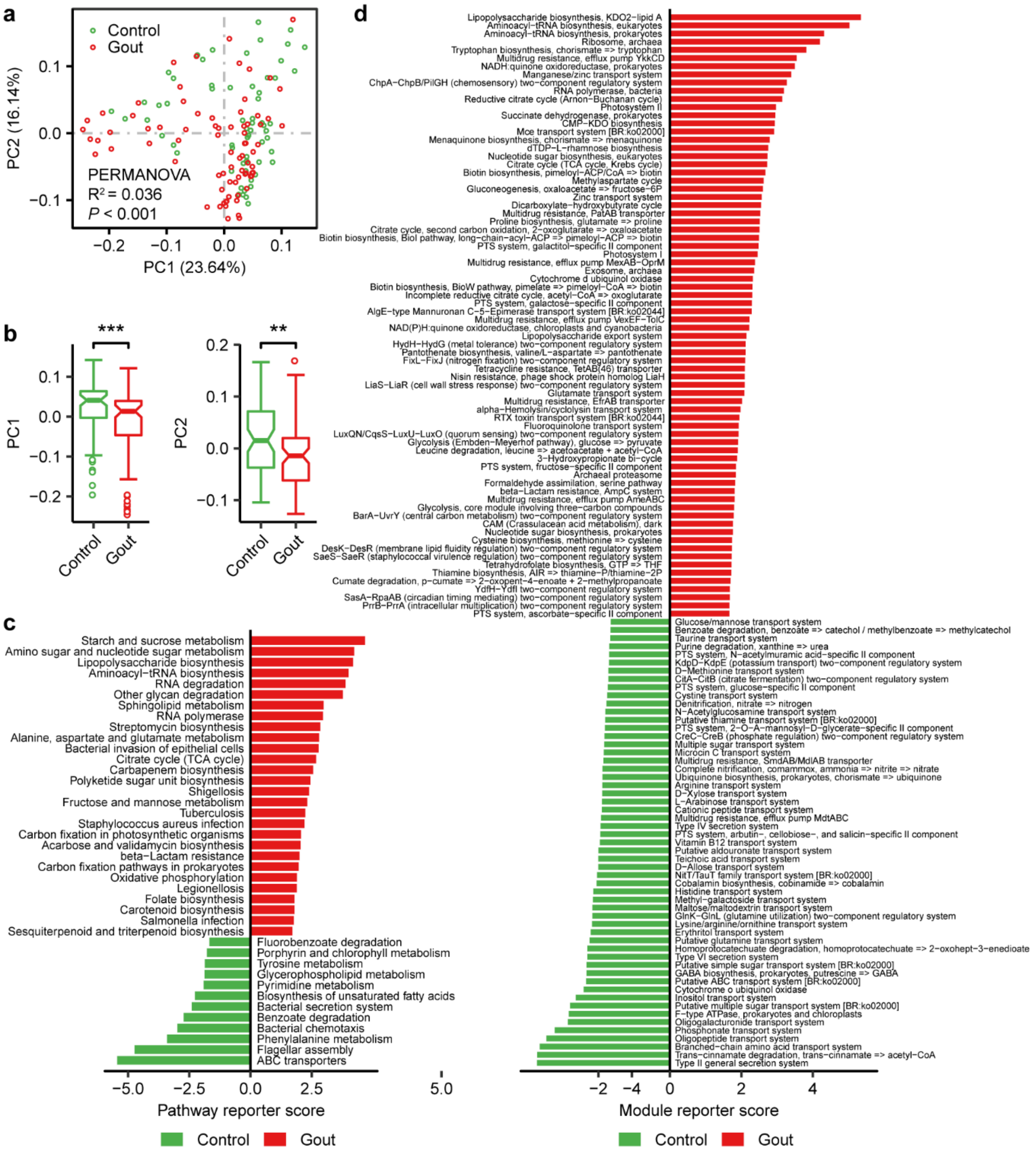

Supplementary Figure 5. Gut microbial gene functions between healthy controls and gout patients in discovery cohort. **(a)** PCA based on the KOs relative abundance profile (green, healthy control, n = 63; red, gout patient, n = 77). PERMANOVA calculation based on the Bray-Curtis distance. **(b)** The distribution of healthy controls and gout patients along PC1 and PC2 were shown on the boxes and whisker plots. Healthy controls were significantly different from gout patients based on Wilcoxon rank-sum test (‘**’, $P < 0.01$; ‘***’, $P < 0.001$). **(c, d)** Different pathways or modules in healthy controls and gout patients. Pathways or modules were shown if reporter score > 1.65 or < -1.65. Green, enriched in healthy controls; red, enriched in gout patients. For all box and whisker plots, the center line represents median. The

bounds of box represent the first and third quartiles. The upper whisker extends from the hinge to the largest value no further than 1.5 * interquartile range (IQR) from the hinge. The lower whisker extends from the hinge to the smallest value at most 1.5 * IQR of the hinge. The notch represents a confidence interval around the median as the median +/- 1.58*IQR/sqrt(n).

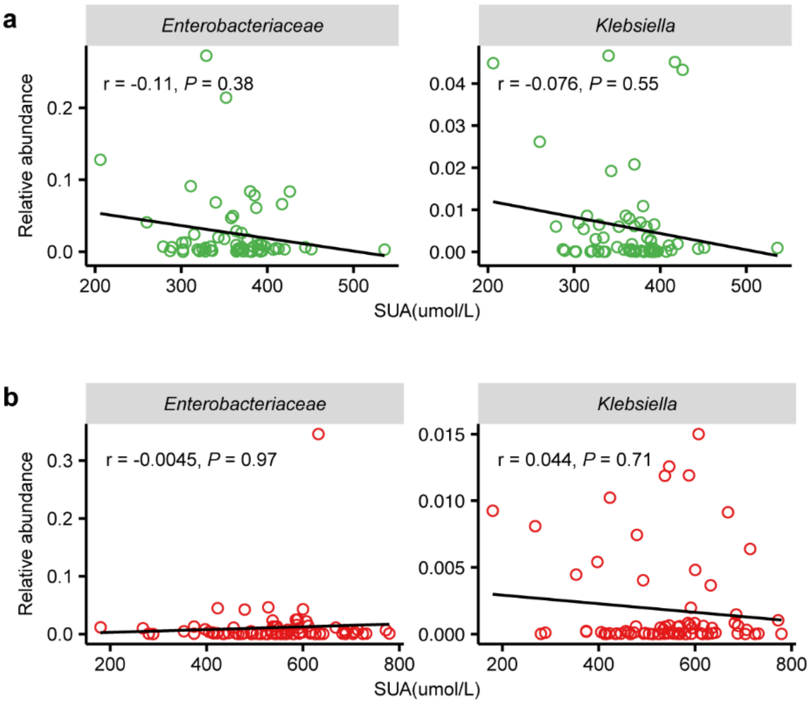

Supplementary Figure 6. The associations for Enterobacteriaceae and Klebsiella with SUA within gout patients and healthy controls.

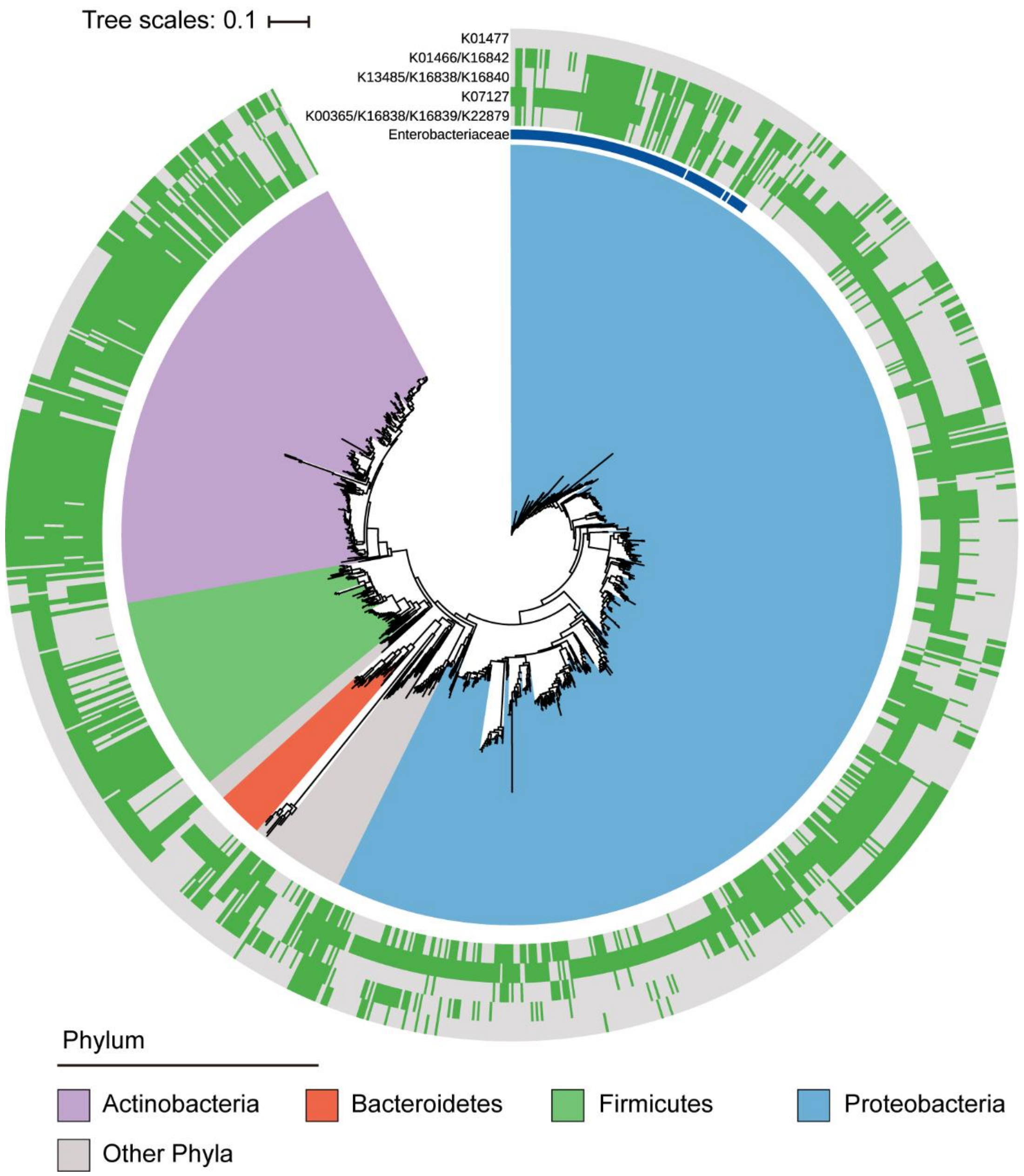

Supplementary Figure 7. Urate degradation gene in bacteria. The tree represents 1,135 species which contain at least one urate degradation gene in KEGG database (v79). Species belonging to *Enterobacteriaceae* were labeled in the middle ring. Green color in the out ring denotes the presence of the genes in the species alongside the tree.

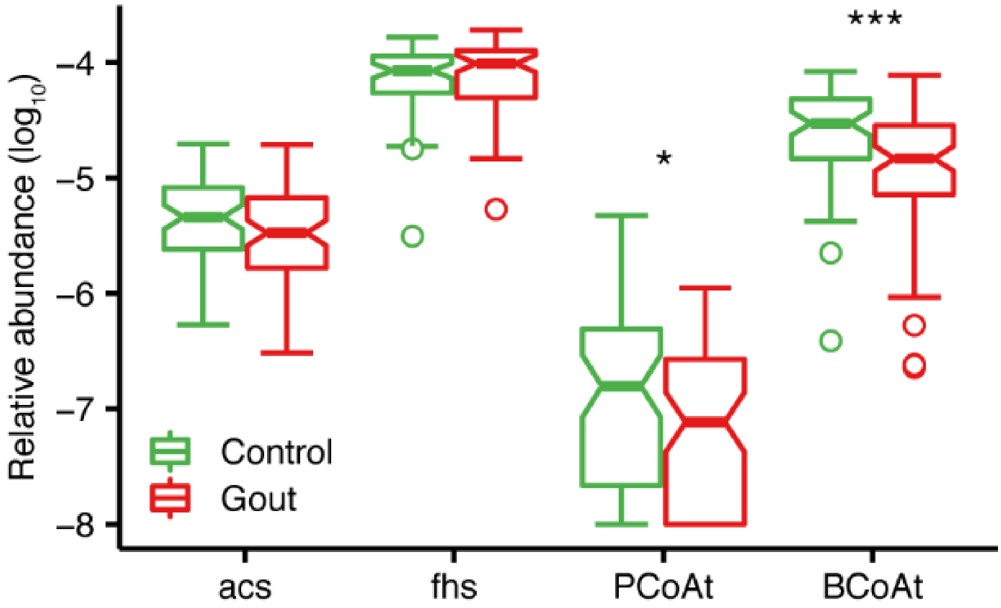

Supplementary Figure 8. Relative abundance of genes related to acetate, propionate and butyrate in healthy controls and gout patients. Wilcoxon rank-sum test was used to determine significance, '*' denotes $P < 0.05$; '**' denotes $P < 0.01$; '***' denotes $P < 0.001$. Abbreviation: acs, O dehydrogenase/acetyl-CoA synthase complex; fhs, formate-tetrahydrofolate ligase; PCoAt, propionate CoA-transferase/propionyl-CoA:succinate-CoA transferase; BCoAt, butyryl-CoA transferase. For all box and whisker plots, the center line represents median. The bounds of box represent the first and third quartiles. The upper whisker extends from the hinge to the largest value no further than 1.5 * interquartile range (IQR) from the hinge. The lower whisker extends from the hinge to the smallest value at most 1.5 * IQR of the hinge. The notch represents a confidence interval around the median as the median +/- 1.58*IQR/sqrt(n).

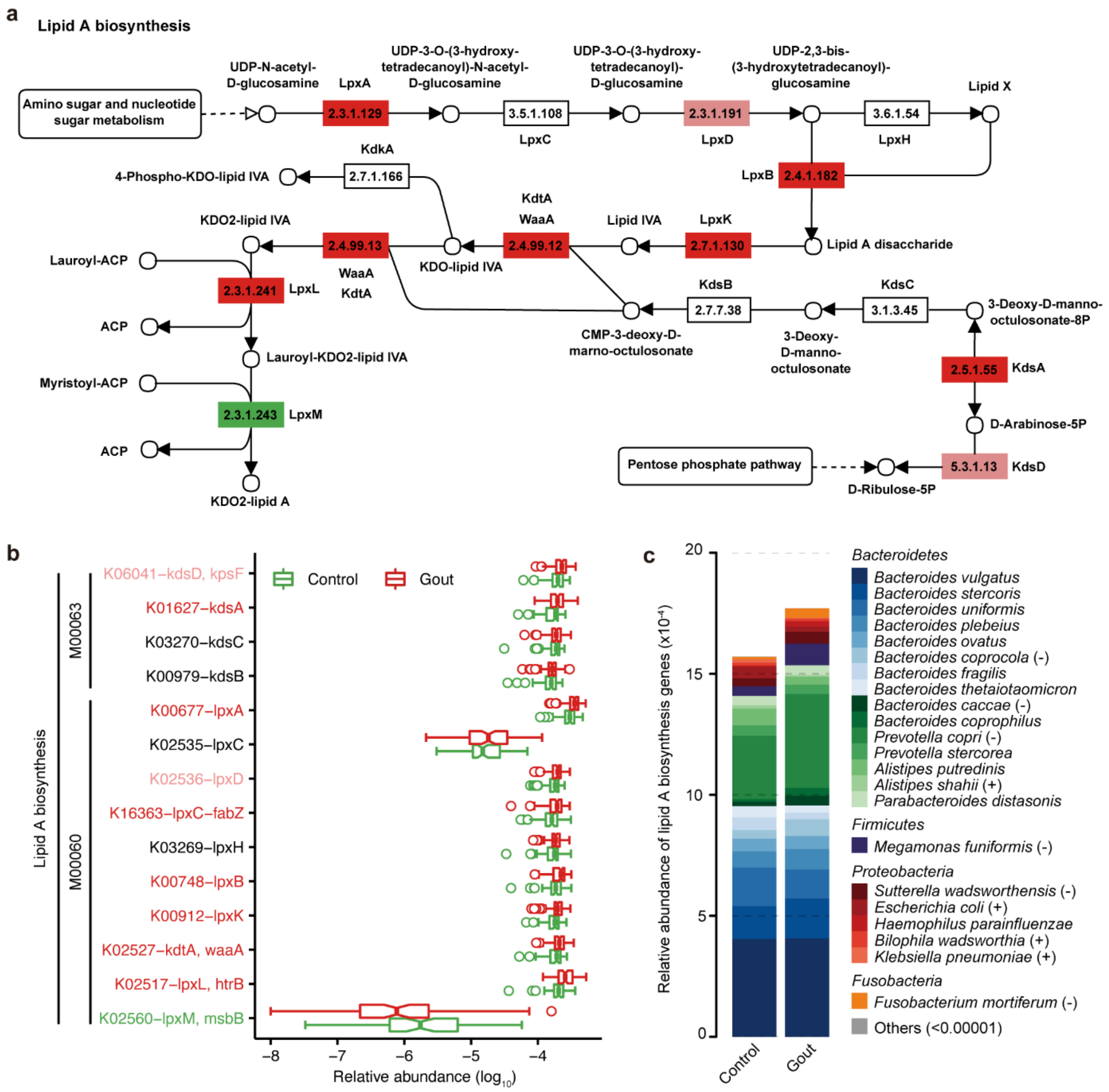

Supplementary Figure 9. Gout-associated microbial genes related to lipid A biosynthesis. **(a)** Module for lipid A biosynthesis. **(b)** Relative abundance of KOs involved in lipid A biosynthesis. Significantly enriched KOs were identified by Wilcoxon rank-sum test and the boxes or KO names were colored according to the direction of enrichment. Green and light green, enriched in healthy control (Green, FDR < 0.05; light green, P < 0.05); Red and light red, enriched in gout patients (Red, FDR < 0.05; light red, P < 0.05); Boxes with no color or KO names with black, no difference; Boxes with grey, not detected in samples. **(c)** Contributions of species to lipid A biosynthesis. Species with mean relative abundance more than 0.00001 in the patient or healthy control group were shown. Species with significantly different relative abundance were marked with '+' or '-' (P < 0.05), which was also corresponding to healthy control-enriched or gout patient-enriched, respectively. For all box

and whisker plots, the center line represents median. The bounds of box represent the first and third quartiles. The upper whisker extends from the hinge to the largest value no further than 1.5 * interquartile range (IQR) from the hinge. The lower whisker extends from the hinge to the smallest value at most 1.5 * IQR of the hinge. The notch represents a confidence interval around the median as the median +/- 1.58*IQR/sqrt(n). Abbreviations: kdsD, kpsF, arabinose-5-phosphate isomerase; kdsA, 2-dehydro-3-deoxyphosphooctonate aldolase; kdsC, 3-deoxy-D-manno-octulosonate 8-phosphate phosphatase; kdsB, 3-deoxy-manno-octulosonate cytidylyltransferase; lpxA, UDP-N-acetylglucosamine acyltransferase; lpxC, UDP-3-O-[3-hydroxymyristoyl] N-acetylglucosamine deacetylase; lpxD, UDP-3-O-[3-hydroxymyristoyl] glucosamine N-acyltransferase; lpxC-fabZ, UDP-3-O-[3-hydroxymyristoyl] N-acetylglucosamine deacetylase / 3-hydroxyacyl-[acyl-carrier-protein] dehydratase; lpxH, UDP-2,3-diacylglucosamine hydrolase; lpxB, lipid-A-disaccharide synthase; lpxK, tetraacyldisaccharide 4'-kinase; kdtA, waaA, 3-deoxy-D-manno-octulosonic-acid transferase; lpxL, trB, Kdo2-lipid IVA lauroyltransferase; lpxM, sbB, lauroyl-Kdo2-lipid IVA myristoyltransferase.

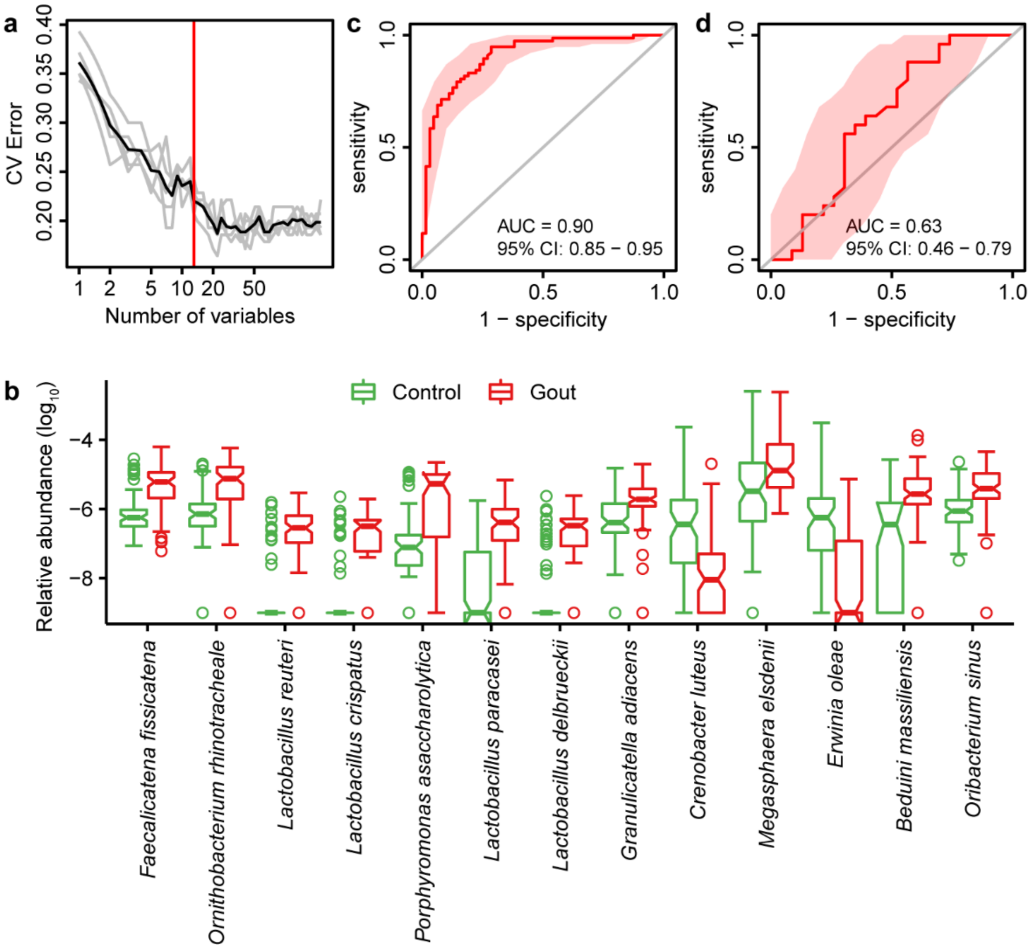

Supplementary Figure 10. Classification of gout by relative abundance of bacterial species. **(a)** Distribution of 5 trials of 10-fold cross-validation error in random forest classification of gout as the number of gene increases. The model was trained using the relative abundance of the genes in discovery cohort. The black curve indicates the average error of the five trials (gray lines). The red line marks the number of gene in the optimal set. **(b)** Relative abundance of 13 bacterial species markers in discovery cohorts. **(c)** Receiver operating curve (ROC) for the discovery samples. **(d)** ROC for the validation samples (healthy control, n = 23; gout patient, n = 25). For all box and whisker plots, the center line represents median. The bounds of box represent the first and third quartiles. The upper whisker extends from the hinge to the largest value no further than 1.5 * interquartile range (IQR) from the hinge. The lower whisker extends from the hinge to the smallest value at most 1.5 * IQR of the hinge. The notch represents a confidence interval around the median as the median +/- 1.58*IQR/sqrt(n).

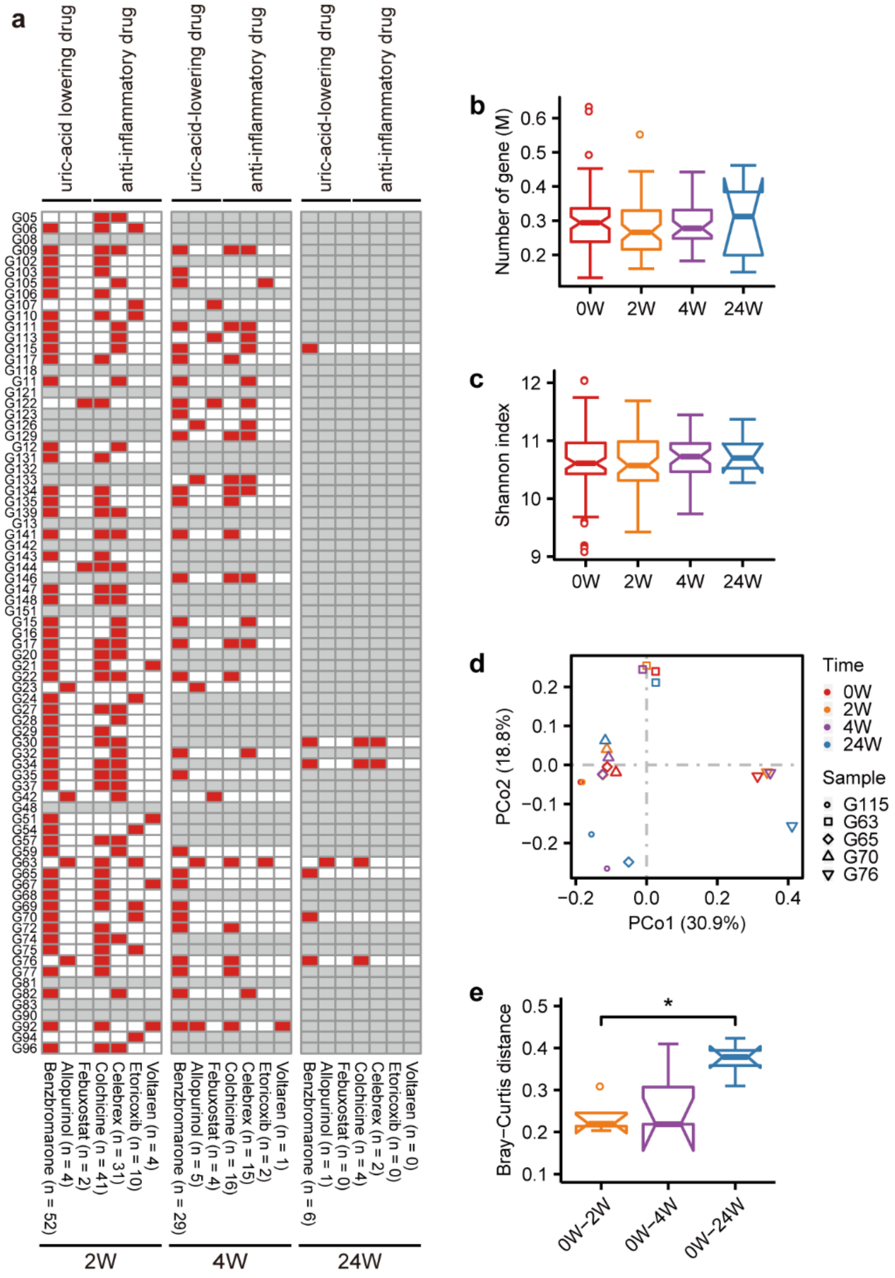

Supplementary Figure 11. Alternation of gut microbiota by therapeutic intervention in gout. **(a)** Medication and sample collection at different time points of 77 gout patients (2W, n = 61; 4W, n = 38; 24W, n = 7). Rows and columns represent individuals and drugs, respectively. Red and white color denote individuals using drugs or not, and gray denotes fecal samples that were not collected for metagenomics sequencing. **(b, c)** Box and whisker plot of gene count (B) and alpha-diversity (Shannon index) (C) in gout patients before and after treatment. **(d)** PCoA based on Bray-Curtis distance at gene level of 5 gout patients whose fecal samples were collected at four time points. **(e)** Box and whisker plot of Beta diversity between before and after treatment of 5 gout patients. Wilcoxon rank-sum test: '*' denotes $P < 0.05$. For all box and whisker plots, the center line represents median. The bounds of box represent the first and third quartiles. The

upper whisker extends from the hinge to the largest value no further than 1.5 * interquartile range (IQR) from the hinge. The lower whisker extends from the hinge to the smallest value at most 1.5 * IQR of the hinge. The notch represents a confidence interval around the median as the median +/- 1.58*IQR/sqrt(n).

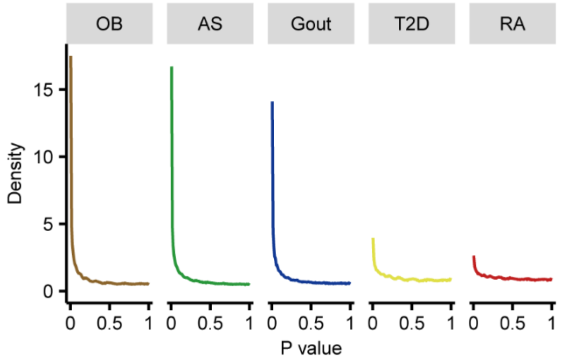

Supplementary Figure 12. The distribution of *P*-values for the differential genes in gout cohort in the presented study as well as four additional public case-control metagenomic datasets. Sample size of OB, AS, T2D and RA was the same as gout with 63 healthy controls and 77 patients, respectively.

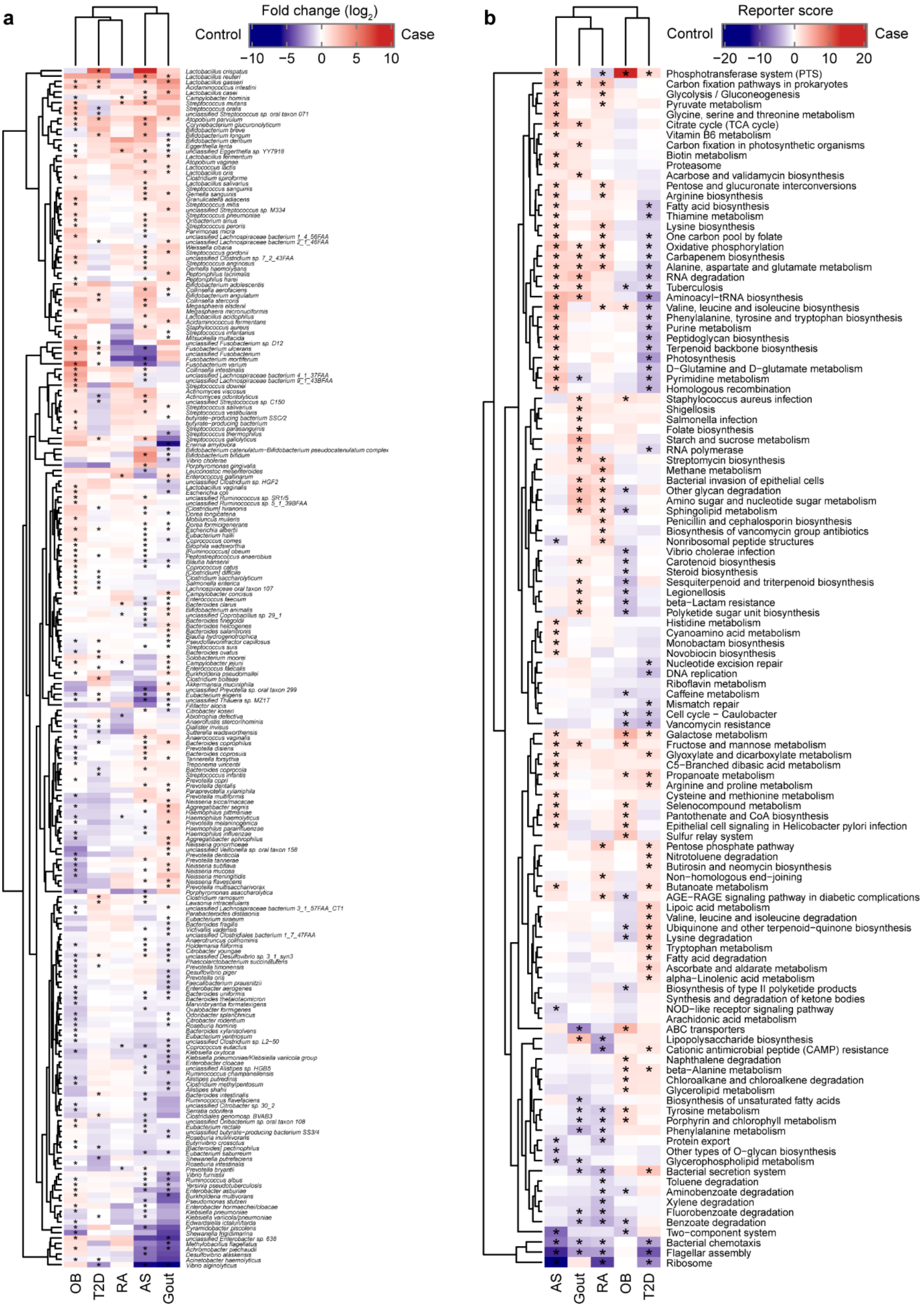

Supplementary Figure 13. Comparison of gut microbial functions and species between gout and other auto-immune and metabolic diseases. **(a)** Comparison of differential species in AS,

gout, OB, RA and T2D. Purple, enriched in healthy controls; red, enriched in patients. **(b)** Comparison of microbial gene functions in AS, gout, OB, RA and T2D. Purple, enriched in healthy controls; red, enriched in patients. ‘*’ denotes reporter score of pathways > 1.65 or < -1.65.

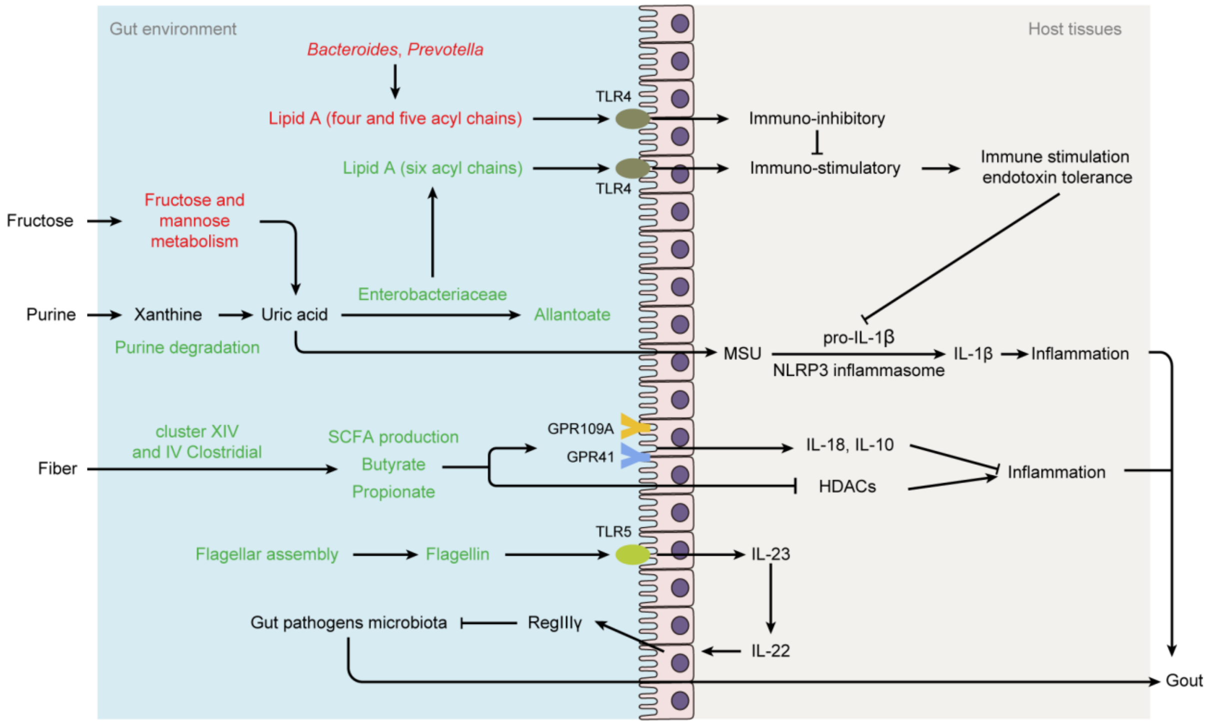

Supplementary Figure 14. A hypothetical model of gut microbes influencing the development of gout based on the findings of the presented study. Green text denotes depleted species or functions in gout patients; red text denotes enriched species or functions in gout patients. Abbreviation: MSU, monosodium urate; TLR4, Toll-like receptor 4; TLR5, Toll-like receptor 5; GPR41, G protein-coupled receptor 41; GPR109, G protein-coupled receptor 109A; HDAC, histone deacetylases; Th17, T-helper 17 cells; IL-1β, interleukin-1β; IL-23, interleukin-23; IL-22, interleukin-22.
