# Supplementary material for: Metagenomic analysis revealed the potential role of gut microbiome in gout
Source: NPJ Biofilms Microbiomes. 2021 Aug 9;7:66. doi: 10.1038/s41522-021-00235-2 (PMC8352958; doi:10.1038/s41522-021-00235-2)
Supplement: Supplementary file 3 — Reporting Summary [file 41522_2021_235_MOESM3_ESM.pdf]

nature portfolio

Corresponding author(s): Qingchun Huang, Zhang Wang, Runyue Huang

Last updated by author(s): 2021/06/09

Reporting Summary

Nature Portfolio wishes to improve the reproducibility of the work that we publish. This form provides structure for consistency and transparency in reporting. For further information on Nature Portfolio policies, see our Editorial Policies and the Editorial Policy Checklist.

Statistics

For all statistical analyses, confirm that the following items are present in the figure legend, table legend, main text, or Methods section.

n/a	Confirmed	
☐	☒	The exact sample size (*n*) for each experimental group/condition, given as a discrete number and unit of measurement
☐	☒	A statement on whether measurements were taken from distinct samples or whether the same sample was measured repeatedly
☐	☒	The statistical test(s) used AND whether they are one- or two-sided<br>*Only common tests should be described solely by name; describe more complex techniques in the Methods section.*
☐	☒	A description of all covariates tested
☐	☒	A description of any assumptions or corrections, such as tests of normality and adjustment for multiple comparisons
☐	☒	A full description of the statistical parameters including central tendency (e.g. means) or other basic estimates (e.g. regression coefficient) AND variation (e.g. standard deviation) or associated estimates of uncertainty (e.g. confidence intervals)
☐	☒	For null hypothesis testing, the test statistic (e.g. *F*, *t*, *r*) with confidence intervals, effect sizes, degrees of freedom and *P* value noted<br>*Give P values as exact values whenever suitable.*
☒	☐	For Bayesian analysis, information on the choice of priors and Markov chain Monte Carlo settings
☐	☒	For hierarchical and complex designs, identification of the appropriate level for tests and full reporting of outcomes
☐	☒	Estimates of effect sizes (e.g. Cohen's *d*, Pearson's *r*), indicating how they were calculated

Our web collection on statistics for biologists contains articles on many of the points above.

Software and code

Policy information about availability of computer code

Data collection	No software was used for data collection.
Data analysis	All analyses performed in this study utilize a set of published software and algorithms with custom codes for secondary data processing and figure visualization. Bioinformatic tools used in this study include SOAPaligner (v2.22), BLAT (v.36), Megablast (v2.2.26), MetaPhlAn3 (v3.0), PyNAST (v1.2.2), FastTree (v2.1.7), BLASTP (v2.2.26) and randomForest R package (4.6-14). The custom codes in this study were implemented in Python (v2.7.15 & v3.6.8) and R (v3.4). The key computer codes for the analyses in this study are available in GitHub (https://github.com/hyf-2021/Gout-script).

For manuscripts utilizing custom algorithms or software that are central to the research but not yet described in published literature, software must be made available to editors and reviewers. We strongly encourage code deposition in a community repository (e.g. GitHub). See the Nature Portfolio guidelines for submitting code & software for further information.

Data

Policy information about availability of data

All manuscripts must include a data availability statement. This statement should provide the following information, where applicable:

- Accession codes, unique identifiers, or web links for publicly available datasets
- A description of any restrictions on data availability
- For clinical datasets or third party data, please ensure that the statement adheres to our policy

The metagenomic shotgun sequencing data for all samples have been deposited in the CNGB Nucleotide Sequence Archive (CNSA) under accession code CNP0000284. Other data that support the findings of this study are available within the paper and its Supplementary Information files or from the corresponding author upon reasonable request.

Field-specific reporting

Please select the one below that is the best fit for your research. If you are not sure, read the appropriate sections before making your selection.

☒ Life sciences ☐ Behavioural & social sciences ☐ Ecological, evolutionary & environmental sciences

For a reference copy of the document with all sections, see nature.com/documents/nr-reporting-summary-flat.pdf

Life sciences study design

All studies must disclose on these points even when the disclosure is negative.

Sample size	Given the exploratory nature of this study, no formal power calculation was performed. This study represents the largest study to understand gut microbiome in gout with a total number of 307 samples from 102 gout patients and 86 healthy controls.
Data exclusions	Patients and healthy controls had to meet the following criteria: (1) 15-69 years of age; (2) no antibiotics and glucocorticoid use within 3 months and 1 month, respectively; (3) no gastrointestinal diseases, such as gastrointestinal surgery, Crohn's disease, ulcerative colitis, or acute diarrhea; (4) no history of severe, progressive or uncontrolled cardiac, hepatic, renal, mental, or hematological disease; and (5) no history of drug abuse.
Replication	This study includes both a discovery and validation cohort. 140 subjects were recruited from 2016-2017 and used as discovery cohort. An additional 48 subjects were recruited in 2018 as validation cohort. The key associations between gut microbiome and gout were validated in the validation cohort. A microbiome-based disease classifier yielded high predictability in discovery and validation cohorts.
Randomization	This is an observational study without treatment groups so randomization is not applicable.
Blinding	This is an observational study without treatment groups so blinding is not applicable.

Reporting for specific materials, systems and methods

We require information from authors about some types of materials, experimental systems and methods used in many studies. Here, indicate whether each material, system or method listed is relevant to your study. If you are not sure if a list item applies to your research, read the appropriate section before selecting a response.

Materials & experimental systems

n/a	Involved in the study	
☒	☐	Antibodies
☒	☐	Eukaryotic cell lines
☒	☐	Palaeontology and archaeology
☒	☐	Animals and other organisms
☐	☒	Human research participants
☒	☐	Clinical data
☒	☐	Dual use research of concern

Methods

n/a	Involved in the study	
☒	☐	ChIP-seq
☒	☐	Flow cytometry
☒	☐	MRI-based neuroimaging

Human research participants

Policy information about studies involving human research participants

Population characteristics	Between May 2016 and September 2018, we recruited 102 male acute gout patients and 86 age-matched male healthy controls, including 77 gout patients (age: 39.9 ± 12.9, BMI: 25.0 ± 2.5) and 63 healthy controls (age: 40.0 ± 12.1, BMI: 23.5 ± 3.0) in discovery cohort and 23 gout patients (age: 41.9 ± 14.4, BMI: 25.8 ± 2.9) and 25 controls (age: 38.3 ± 13.6, BMI: 22.4 ± 2.3) in validation cohorts.
Recruitment	Patients were diagnosed with gout as determined by the 2015 ACR/EULAR classification criteria and suitability for the treatment in this study. 140 subjects were recruited from 2016-2017 and used as discovery cohort. An additional 48 subjects were recruited in 2018 as validation cohort. For discovery cohort, after collecting fecal samples at baseline, gout patients were treated with uric-acid-lowering (benzbromarone, allopurinol, febuxostat) and anti-inflammatory drugs (colchicine, celecoxib, etoricoxib, betamethasone, voltaren), and fecal samples were collected after drugs treatment for 2 weeks (2W, n = 70), 4 weeks (4W, n = 40), and 24 weeks (24W, n = 9).
Ethics oversight	This study was approved by the Medical Ethics Committee of the Second Affiliated Hospital of Guangzhou University of Chinese Medicine (B2016-103-01). All participants provided written informed consent.

Note that full information on the approval of the study protocol must also be provided in the manuscript.
